# Machine Learning Approach for Predicting Older Adults’ Responsiveness to Cognitive Training Interventions: Data from the ACTIVE Study

**DOI:** 10.3390/jintelligence14040056

**Published:** 2026-04-01

**Authors:** Petra Vargek, Sašo Karakatič, Karin Bakračevič

**Affiliations:** 1 Department of Psychology, Faculty of Arts, University of Maribor, 2000 Maribor, Slovenia; petra.vargek@sbvzt.hr; 2 Special Hospital for Medical Rehabilitation Varaždinske Toplice, 42223 Varaždinske Toplice, Croatia; 3 Intelligent Systems Laboratory, University of Maribor, 2000 Maribor, Slovenia; saso.karakatic@um.si

**Keywords:** cognitive training, older adults, transfer, machine learning, responsiveness, personalized cognitive training, ACTIVE study, magnification effect

## Abstract

In recent years, there has been increasing interest in personalizing cognitive training to enhance the likelihood of positive training effects at the individual level. Machine learning methods have proven suitable for this purpose due to their ability to generate predictions at the individual level. The aim of the study was to develop supervised machine learning models to predict near and far transfer of three cognitive training interventions (memory training, reasoning training and speed-of-processing training) based on baseline characteristics of elderly individuals including sociodemographic data, measures of cognitive and everyday functioning and depressive symptoms. In addition, near-transfer models were further utilized to predict individual responsiveness to all three types of cognitive training. Publicly available data from the ACTIVE study were used, which examined the effects of memory training, reasoning training and speed-of-processing training in healthy adults. Multiple supervised machine learning classification algorithms were applied to establish optimal predictive models for each type of cognitive training and transfer measure. Selected models for predicting near transfer were then used to estimate individual responsiveness to all three interventions. The results show selected models for all three types of cognitive training and both near- and far-transfer outcomes demonstrated better discriminative ability than chance based on all included features (AUC range 0.56–0.74), although models predicting far transfer demonstrated limited performance. Predicted responsiveness to cognitive training varied according to participant characteristics. Differences between model-predicted responders indicate that initially advantaged participants would have greater likelihood of benefiting from a broader range of interventions compared to initially disadvantaged ones, which would support magnification effects. The developed models need external validation, but have practical potential for selecting effective interventions tailored to individual characteristics, which could improve the future implementation of cognitive training programs.

## 1. Introduction

Cognitive training (CT) has gained significant attention for its potential to mitigate age-related cognitive decline, particularly by targeting domains most vulnerable to deterioration, such as memory, reasoning and processing speed. The results of the research carried out so far regarding the efficacy of cognitive training vary, which is at least partly due to the heterogeneity of the interventions and the different measures of efficacy of intervention (Green et al., 2019; von Bastian & Oberauer, 2014). Studies show that while the effects of cognitive training on measures similar to those that are trained, i.e., near-transfer effects, provide reliable small-to-medium effects, the effects on broader measures, i.e., far-transfer effects on different cognitive functions than those trained, or measures of everyday functioning, tend to be smaller and less consistent (Basak et al., 2020; Gavelin et al., 2020; Melby-Lervåg et al., 2016; Sala et al., 2019; Teixeira-Santos et al., 2019). Considering that cognitive training is time consuming and requires considerable cognitive engagement, which is accompanied by problems with adherence to the training (Turunen et al., 2019), it can be discouraging when the intervention is ineffective, especially in practical circumstances.

In recent years there have been attempts to personalize cognitive training to maximize the probability of positive effects for the specific individual. Examining individual trajectories of learning and individual differences in response to cognitive training could enable a more personalized cognitive training experience and provide insights regarding the inconsistency of the results of cognitive training studies (Feng et al., 2023; Rennie et al., 2021; Shani et al., 2019; Smid et al., 2020; Traut et al., 2021).

Studies show that individual differences are predictive of the effects of cognitive training, yet the direction of the effects is still not entirely clear. Magnification effects are a demonstration of greater improvement following cognitive training in participants with initial high cognitive functioning (Foster et al., 2017; Fu et al., 2020; Guye et al., 2017). Nonetheless, a compensation effect is also present, revealing greater improvement following intervention in participants with initial low cognitive ability (Karbach et al., 2017; Traut et al., 2021). Some studies show that these opposite effects could be the function of the cognitive domain, the training approach and/or the type of the effects being analyzed (Bruno et al., 2024; Karbach et al., 2017; Shaw & Hosseini, 2021). In addition, effects can be impacted by the characteristics of the participants, such as age, years of education, beliefs or other factors that are, perhaps, not being examined (Bürki et al., 2014; Jaeggi et al., 2014; Karbach et al., 2017; Lövdén et al., 2012; Shaw & Hosseini, 2021). Empirical evidence altogether indicates a complex relationship between the initial characteristic and the effects of the intervention.

Cognitive training is theoretically grounded in concepts like cognitive plasticity (Baltes & Lindenberger, 1988; Park et al., 2007) and cognitive reserve (Stern et al., 2020), and considering those theories, it has to be sufficiently challenging for individual to elicit change. These processes of change could be further enhanced with personalization of training and utilizing more advanced, machine learning methods.

Shani et al. (2019) describe the process of personalization of cognitive training. First, prior to the cognitive training intervention, personal characteristics (age, cognitive abilities, personal traits, etc.) are collected. The second part the training selection is carried out based on the individual characteristics and a machine learning (ML) algorithm. The third part of the process is continual training adaptation, according to the subject’s baseline characteristics and performance in training, in order to achieve optimal performance.

Novel methodological approaches, such as machine learning methods, appear to be ideal for personalizing cognitive training, because they can provide predictions at a single subject level (Orrù et al., 2020). They are suitable for analyzing complex relationships and identifying subtle patterns in the dataset. In line with this, there is a higher tolerance for multidimensionality of data and assumptions violations (about linearity, etc.), which are problematic in classical statistical methods (Coutanche & Hallion, 2020). Proposing minimal assumptions, they are strongly focused on achieving the best prediction accuracy, even at the cost of the interpretability of a given solution (Orrù et al., 2020; Yarkoni & Westfall, 2017).

As opposed to traditional statistical methods that evaluate the model based on how well it explains the patterns in an original dataset, machine learning techniques allow us to test whether a model can generalize to new data (Coutanche & Hallion, 2020). The established procedure of these machine learning techniques is to first apply a machine learning algorithm for describing patterns and relationships on one dataset, described as a “training” dataset, and then to validate the model on an independent, “testing” dataset (Coutanche & Hallion, 2020). In order to provide more accurate predictions based on the new dataset, cross-validation can additionally be performed on the training dataset for selection of optimal parameters (Orrù et al., 2020).

The application of machine learning or, more broadly, artificial intelligence methods for the purpose of personalizing cognitive training, is at an early stage of development (Adolphe et al., 2025). Most research has examined task adaptivity as an alternative to traditional adaptive algorithms (Adolphe et al., 2025). While traditional adaptive tasks primarily rely on recent task performance, newer approaches incorporate multidimensional data to enable more personalized task adaptation (Adolphe et al., 2025), including both past and recent task performance (Sandeep et al., 2020) or broader data collected through cognitive test batteries (Graessel et al., 2024). Several studies that apply machine learning methods have examined individual characteristics to analyze learning trajectories during cognitive training or to predict cognitive training outcomes. A recent study (Feng et al., 2023) has shown that learning trajectories in working memory training in undergraduate students can be grouped and predicted by the combination of different individual characteristics. Similarly, one study (Rennie et al., 2020) has identified subgroups of children with differential responses to cognitive training predicted by fluid intelligence. Another study on children (Vladisauskas et al., 2022) used supervised machine learning algorithms to predict the individual effectiveness of executive function cognitive training considering baseline individual differences in executive functions and attentional abilities.

### Study Aims and Research Questions

With the exception of commercial cognitive exercise applications that offer intervention with personalized selection primarily based on user preferences and the initial evaluation of the user’s strengths and weaknesses (Bedoya & Polanco, 2025; Peretz et al., 2011; Shani et al., 2019; Shatil et al., 2010), the scientific literature does not provide solutions for cognitive training personalization grounded in objective decision making regarding the selection of an appropriate type of cognitive training for an individual based on their baseline characteristics.

The aim of this study was to develop machine learning models to predict the efficacy of various training types and then utilize those models to predict individual responsiveness across all training interventions to enable appropriate intervention selection for that individual.

Accordingly, the first research question was as follows: can supervised machine learning models predict the near and far transfer effects of different cognitive training interventions (memory, reasoning and speed-of-processing training) in healthy older adults based on their baseline characteristics, including sociodemographic factors, depressive symptoms, cognitive measures and everyday functioning?

The second research question was as follows: can we utilize established models of near transfer for each type of cognitive training to predict individual responsiveness to all cognitive training interventions?

The third question was as follows: do elderly individuals with different predicted responsiveness differ in their individual characteristics, specifically, cognitive profiles, measures of cognitive and everyday functioning, depressive symptoms and sociodemographic data?

Previous ML research was primarily focused on predicting outcomes of a single specific intervention like the learning trajectory in training of working memory (Feng et al., 2023) or near-transfer effects of executive functions training (Vladisauskas et al., 2022). Our study extends this framework to encompass a broader range of cognitive interventions enabling cross-intervention prediction and including not only near- but also far-transfer outcomes. Cross-intervention prediction facilitates the comparison of potential responsiveness to different cognitive training interventions at the individual level. By that, we could estimate how many and which type of cognitive training may have the greatest potential for a given person and identify their individual differences. This approach represents a step toward personalized cognitive training interventions in healthy older adults, enabling more precise targeting of effective intervention.

## 2. Materials and Methods

### 2.1. Data

The subset (pre- and post-training datasets only) of publicly available data from the longitudinal study Advanced Cognitive Training for Independent and Vital Elderly (short: ACTIVE study) was acquired.

This is one of the largest randomized clinical trials, carried out in the period from 1999 to 2008, which examined the effects of different cognitive training interventions (memory, reasoning or speed-of-processing training) on healthy older adults. The detailed methodology, as well as the aims of the study and its major findings, are available elsewhere (K. Ball et al., 2002; Jobe et al., 2001; Tennstedt & Unverzagt, 2013). In our study, we applied different supervised machine learning algorithms to build and test predictive models of the effects of cognitive training, which were then used to differentiate subjects with regard to their predicted responsiveness to various cognitive training interventions.

#### 2.1.1. Participants

The participants in the ACTIVE study were community-dwelling elderly participants recruited throughout six metropolitan regions across the United States (University of Alabama at Birmingham, Hebrew Rehabilitation Center for Aged in Boston, Indiana University, Johns Hopkins University, Wayne State University and Pennsylvania State University). Recruitment was conducted through driving registries, residents of senior housing and residential facilities, users of senior centers and organizations, churches, health clinics and various local programs and community organizations. The targeted population was elderly individuals at risk of functional decline who are living largely independent of formal care at the beginning of the study. Accordingly, eligibility for participation was restricted to people 65 years or older with no major health problems, who were living largely independently and had no conditions that could interfere with cognitive training participation. Excluded were subjects with impaired cognition (MMSE score less than 23 (Folstein et al., 1975)), with sensory (vision/hearing) or communication impairment (self-reported and/or by interviewer’s rating), deficits in activities of daily living (self-reported extensive assistance with personal hygiene, bathing or dressing), and with self-reported diagnosis of Alzheimer’s Disease, stroke (in the previous 12 months) or cancer. Five thousand elderly individuals were contacted for participation, out of which 2832 were eligible for participation and 30 of those who were eligible were randomized inappropriately, thus were excluded from analyses (see details: K. Ball et al., 2002). The resulting sample consisted of 2802 individuals (Jobe et al., 2001), who were randomized to one out of three cognitive training interventions or a passive control group.

#### 2.1.2. Study Design and Intervention

The ACTIVE study design was a randomized controlled trial with a four-group design, including three intervention groups and one passive control group. The interventions consisted of three types of cognitive training, and each intervention group received training for one out of three cognitive abilities: memory, inductive reasoning or speed of processing. The interventions were conducted by certified trainers in small groups and consisted of ten sessions for two or three times per week, with a duration of 60–75 min, and all sessions were received within a six-week period. In all intervention groups, the first five sessions were focused on learning and practicing strategies, while the remaining five sessions provided only practice, without learning new strategies (K. Ball et al., 2002; Jobe et al., 2001).

For building the supervised machine learning models in our study, we used only the cognitive training intervention subsets of data: memory cognitive training (N = 703), inductive reasoning cognitive training (N = 699), and speed-of-processing cognitive training (N = 702). The included samples in our study were participants who completed initial cognitive training intervention and had sufficient pre-test and post-test data needed for the calculation of training effects. The final number of valid cases with training effects (class values) were N = 641 for the memory training, N = 629 for the reasoning training, and N = 643 (near transfer) and N = 653 (far transfer) for the speed-of-processing training. For the clustering of cognitive profiles and for predicting the responsiveness to different cognitive training interventions, the whole dataset (N = 2802) was used, including the experimental groups and the control group data (N = 698).

#### 2.1.3. Materials

The following pre- and post-intervention measures were used:

- General cognitive functioning: Mini-Mental State Examination (MMSE) score (Folstein et al., 1975).
- Measures of specific cognitive abilities: three memory measures: the Hopkins Verbal Learning Test (HVLT) (Brandt, 1991), Rey Auditory–Verbal Learning Test (AVLT) (Rey, 1941) and Rivermead Behavioral Memory Test immediate recall (RVM) (Wilson et al., 1985); three reasoning measures: Letter Series (LS) (Thurstone & Thurstone, 1949), Letter Sets (LT) (Ekstrom et al., 1976) and Word Series (WS) (Gonda & Schaie, 1985); and four speed-of-processing measures: subtests of Useful Field of View (UFOV) (Owsley et al., 1998).
- Measures of daily functioning: three subjective measures of daily functioning from the Minimum Data Set for Home Care (MDS) (Morris et al., 1997), including results in subscales: (a) performance of instrumental activities of daily living (IADL), (b) performance in basic ADL and (c) perceived degree of difficulty in completing ADL; and one performance-based measure of daily functioning: Everyday Problems Test (EPT) (Willis et al., 1998).
- Measure of depressive symptoms: Center for Epidemiological Studies—Depression (CES-D) (Radloff, 1977).

Sociodemographic data, including age, gender, and years of education, were used.

### 2.2. Predictive Models

Three separate datasets were used for developing the models: the memory cognitive training dataset, the reasoning cognitive training dataset and the speed-of-processing cognitive training dataset. Two models with different classes were used for each dataset, including near-transfer effect and far-transfer effect, and the set of proposed features was the same in all models.

#### 2.2.1. Features

Sociodemographic variables (age, gender and years of education), pre-test measure of depressive symptoms and all pre-test cognitive and daily functioning measures were included in the predictive models as features. HVLT and AVLT test results had around 10% of missing cases across the training groups, while other features had 1% or less or no cases with missing values. The missing values in HVLT and AVLT were replaced by standardized (z-score) mean result on other memory test(s) that were not missing for that participant), while cases with missing values in other features were handled by respective algorithms. Naïve Bayes ignores a missing value of attribute of given instance, Random Forest distributes instances with missing values across tree branches according to probabilities, while Logistic Regression, Support Vector Machines and Multilayer Perceptron replace missing values with mode/mean of the training dataset. Before entering the model, all features were inspected for collinearity to exclude unnecessary features and to lower the probability of the model overfitting as a result of too many features. If two features correlated higher than 0.7, one of the features was excluded from the model.

#### 2.2.2. Classes

Measures of specific cognitive ability were used to define classes, with a comparable number of measures across domains, consisting of three memory measures (HVLT, AVLT, RVM), three reasoning measures (WS, LS, LT) and four speed-of-processing measures (UFOV 4 subsets). Similar to Vladisauskas et al. (2022) who used the Reliable Change Index (RCI) (Jacobson & Truax, 1991) for classification who improved or not following cognitive training, we used this index for an estimation of effects of the interventions. Among possible variations, the following formula for RCI was implemented (Estrada et al., 2019):

$$RCI=\frac{D_{i}}{\sqrt{{\left({SD}_{Pre}\sqrt{1-R_{PrePost}}\right)}^{2}+{\left({SD}_{Post}\sqrt{1-R_{PrePost}}\right)}^{2}}}$$

where *D_i_* is the individual pre-post difference; *SD_Pre_* and *SD_Post_* are standard deviations at pre-test and post-test, respectively; *R_PredPost_* is internal consistency estimated using Cronbach’s alpha (Cronbach, 1951).

RCI for each measure was calculated using pre- and post-test results and the threshold was set on 1.96. This threshold reflects statistically significant differences on an individual level (compared to a random measurement error alone) with a Type I error rate of 0.05. Correspondingly, if RCI was higher than 1.96, reliable change (i.e., positive effect) was considered; if a result was between −1.96 and 1.96, there was no reliable change; and if the result was lower than −1.96, the negative effect of cognitive training was considered for that specific cognitive ability measure.

The positive sums of RCIs were counted individually for the effects of three cognitive domains: memory, reasoning and processing speed to capture reliable domain improvement considering multiple indicators of the same construct, i.e., cognitive function. The positive sum of memory measures was classified as the near-transfer effect for congruent cognitive training, i.e., memory training. Similarly, near-transfer effects of reasoning/speed-of-processing training were classified by sums in congruent cognitive measures. Far-transfer effects were positive sums in non-congruent cognitive measures, e.g., for memory training, a positive sum of reasoning and speed-of-processing training, etc. Because the primary interest was participants who benefited from intervention, variables were dichotomized into “responsive” or “not responsive”. Therefore, in all calculations, zero or negative sums were classified as “not responsive” reflecting the absence of reliable improvement, rather than equivalence between no change and negative effect, especially given that participants were elderly in whom age-related cognitive decline can occur over time. The results of RCI calculations are presented in Table S1 in Supplementary Materials.

#### 2.2.3. Machine Learning Algorithms

In order to compare and establish optimal models (Jiang et al., 2020) in accordance with the complexity of the relationships between variables, several supervised machine learning algorithms for classification, from simple to more complex, were used: (1) Naive Bayes, (2) Logistic Regression, (3) Multilayer Perceptron, (4) Support Vector Machines, (5) Random Forest; as well as the Ensemble method, combining all five classifiers using a Voting method based on the average of probabilities (soft voting).

According to recommendations from Orrù et al. (2020), methodology that an combines 80/20 train–test split odd data and cross-validation (CV) was applied. A random stratified train–test split was carried out, dividing all three datasets into train (80% of the sample) and test (20% of the sample) datasets. All features were standardized to have zero mean and unit variance. Class imbalance was addressed using instance reweighting (Weka’s ClassBalancer), which assigns weights, so each class has equal total weight in the training set, without changing sample size. This was preferred over resampling methods (e.g., SMOTE) to avoid generating synthetic instances that could introduce artifacts in cognitive assessment data. Feature selection was performed using the Information Gain Attribute Evaluator with the Ranker method to identify relevant features in the model. A ten-fold CV was conducted to assess the performance of each model. Preprocessing steps (class reweighting, feature selection) were performed within each cross-validation fold using training-fold data only.

Hyperparameter tuning was conducted using grid search over a predefined set of parameter combinations for Multilayer Perceptron, Support Vector Machines and Random Forest (Table 1), while for Logistic Regression and Naïve Bayes, default parameters were used. Hyperparameter tuning was performed only on training folds within CV, then the final model was evaluated on the holdout test set. The chosen models were subsequently evaluated and the best performing model among the six was selected for each type of cognitive training and type of transfer effect, taking into account several performance measures: accuracy, true-positive rate, false-positive rate, precision, recall, F1-score, Brier score and area under curve (AUC). AUC quantifies a discriminative ability of model in predicting a binary event by which values range from 0.5 to 1, with higher values representing better discriminative ability. They can be approximately compared to more familiar effect size measures, whereby a weak effect (d = 0.2) corresponds to an AUC value of 0.56, moderate effect size (d = 0.5) corresponds to an AUC value of 0.64, while a large effect size (d = 0.8) corresponds to an AUC value of 0.71 (Pencina et al., 2012).

Model selection (including algorithm comparison and hyperparameter tuning) was performed using cross-validation on the training set only. The hold-out test set was used once for final performance estimation of the selected model.

Based on performance, the best predictive models were selected for both near-transfer effects and far-transfer effects for each of the three cognitive training interventions. The best predictive performance models for near-transfer effects were used to predict the responsiveness of the participants to the cognitive training interventions.

We, therefore, applied all three of the constructed models to the complete dataset, where feature values were known, but the class labels were not. The models were used to predict unknown class labels based on the provided feature values. The methodology workflow is presented in Table 2, and more elaborately in the flowchart in Supplementary Materials (Figure S1).

### 2.3. Cluster Analysis

Cognitive profiles of the participants were created by using cluster analysis. Standardized measures of specific cognitive abilities were included as attributes. The Simple K-means method was applied with K means ++ initialization and Euclidean distance for instances comparison. The number of clusters was decided by the elbow method and a theoretical interpretation of the solutions. We aimed to select a clustering solution that will serve for the interpretation of predicted responsiveness in the context of magnification and compensation effects. Accordingly, we were especially interested in cognitive profiles representing high-cognitive-functioning and low-cognitive-functioning individuals.

### 2.4. Statistical Analysis

Statistical analyses were used to examine whether participants with different responsiveness to various cognitive training types differ in sociodemographic characteristics, depressive symptoms, on measures of cognitive and daily functioning, and in cognitive profiles. The Kolmogorov–Smirnov test was used for testing the normality of the distribution of interval variables. Since all interval variables statistically significantly differ from the normal distribution, the nonparametric Kruskal–Wallis test was used to test the difference in categorial variable by interval variables, with eta-square (η^2^) as a measure of effect size. For post hoc analysis, the Mann–Whitney test with the r as a measure of effect size. The differences in independent categorical variables were tested with chi-square and Cramer’s V as a measure of effect size. For post hoc analysis, observed and expected proportions were statistically tested considering Bonferroni correction, with adjusted residuals as indicators of effect size. For dependent categorical variables, differences were tested using Cochran’s Q, with maximum-corrected η^2^ as effect size. Additionally, McNemar’s test was used for post hoc statistical significance analyses and Cohen’s g as a measure of effect size. For testing statistical significance of distributions on one categorial variable, chi-square was applied with residuals as indicators of effect size. In all post hoc analyses, Bonferroni correction was computed to determine statistical significance cut-off.

All machine learning analyses were performed in the Weka 3.8.6 software package. Descriptive and statistical analyses were performed in IBM SPSS Statistic 20 software.

## 3. Results

Sociodemographic characteristics of participants excluded from the analysis due to missing data class labels differ minimally from those included. Excluded participants were on average slightly older (74.14 ± 6.55 years compared to 73.58 ± 5.84), had fewer years of education (13.16 ± 2.86 compared to 13.56 ± 2.69) and a greater proportion were women (78.8% compared to 75.6%). Distribution of sociodemographic characteristics regarding missing values by intervention group is presented in Table 3^1^. Nonetheless, the differences between the groups considering missing values were not statistically significant.

### 3.1. Predictive Machine Learning Models

In the subgroups for all three cognitive training interventions, Letter Series and Word Series test results correlated higher than 0.7 (cut-off value for high correlation); therefore, Letter Series was excluded from all further analyses, as it had higher correlation with the other reasoning measure (Letter sets) (see correlation matrix of features in Supplementary Materials Table S2). Feature descriptives by datasets are shown in Table 4.

In the feature selection step, we applied the Info Gain Attribute Evaluator using the Ranker method within 10-fold CV on train datasets and performed the ablation method by cumulatively excluding features in models from lowest to highest rank ones. Ranking of the feature importance and ablation results for each selected model are presented in Supplementary Materials (Tables S3–S8). Ranking of the features differed considering specific models. Nonetheless, EPT was among the highest ranking in several models, particularly in memory training models, and in models for far-transfer speed-of-processing training and near-transfer reasoning training. MMSE, age and years of education were consistently in average ranking among models. Measures of speed of processing (UFOV) were often highly ranked, particularly in near transfer speed-of-processing training model, although some UFOV measures appeared among least important features in memory models, especially for near transfer. Gender consistently appeared to have the lowest rank in all models. When feature ablation of individual features was performed, models typically showed deterioration regarding performance metrics. Regarding selected models, small improvement was observed only for the far-transfer reasoning model when gender and Word Series were excluded. However, the improvement was minimal, therefore, features were retained for all cognitive training intervention models, including both outcome classes, near transfer and far transfer.

**Figure 1:**
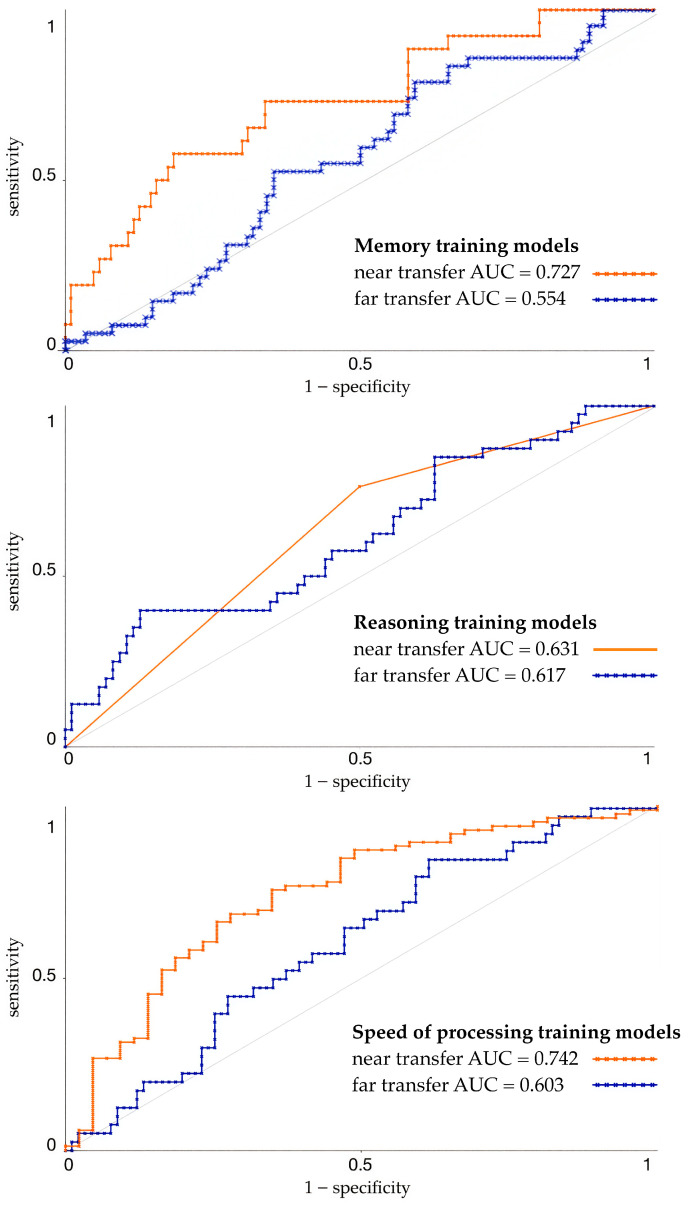
Roc curves for best performing models.

Among six machine learning algorithms, models with best performance for each type of cognitive training and transfer effect were selected. All chosen models for each intervention and for both near transfer and far transfer demonstrated discrimination above chance (Figure 1).

For near-transfer prediction in memory training, the best performing model was an ensemble of models correctly predicting the effect of memory intervention in 66.7% (AUC = 0.727) of cases (weighted average model performance) (Table 5). If we inspect the confusion matrix (Table 6), there is a class imbalance with responsive individuals being in the minority class (20% of cases). Focusing interpretation on responsive class as our target group, model accurately identified most of truly responsive individuals (recall = 0.692). Despite a relatively high ability to detect truly responsive individuals, the precision of model was low (0.34), reflecting a high false-positive rate resulting in a modest balance between precision and recall (F1-score = 0.456).

The accuracy of the prediction of the best-performing model (Naive Bayes) of far transfer in memory intervention was 57.4% (AUC = 0.554) (weighted average model performance) (Table 5). Responsive individuals were minority class (33%) (Table 7). Model accurately identified only 23.8% of individuals truly responsive to intervention (recall = 0.238) and had low precision (0.303), resulting in overall poor performance in predicting responsive class (F1-score = 0.267).

The best-performing model for predicting near transfer in reasoning training was Support Vector Machines, having a weighted average accuracy of 66.7% (AUC = 0.631) (Table 8). Considering class imbalance, responsive subjects were the majority group (63%) (Table 6). Model demonstrated satisfactory performance in prediction of responsive class accurately identifying 76.3% of truly responsive participants (recall = 0.763) with the precision of 0.726, and F1-score of 0.744.

Random Forest was selected as best-performing model for predicting far transfer in reasoning training having weighted average accuracy of 63.5% (AUC = 0.617) (Table 8). Our target group was minority class (32%) (Table 7). Overall performance for predicting responsive class was relatively low (F1-score = 0.410), and it accurately identifies 40% truly responsive subjects with precision of 0.421.

Based on performance metrics, we selected the best models for predicting the effects of the speed-of-processing cognitive training (Table 9). For the best-performing model, Random Forest had a 70.5% average weighted accuracy (AUC = 0.742) in predicting near transfer. Responsive group constituted majority class (67%) (Table 6) and model demonstrated good performance in predicting this class (F1-score = 0.776) with relatively high and well-balanced true-positive rate (0.767) and precision (0.786).

The best performing model for predicting far transfer in the speed-of-processing training was Logistic Regression and had an average weighted accuracy of 54.2% (AUC = 0.603). When focusing on the responsive group, which was a minority class (31%) (Table 7), the model demonstrated a recall of 67.5%, indicating that most truly responsive subjects were correctly identified. The precision was relatively low (0.365), reflecting a high number of false-positive cases resulting in a modest overall performance in predicting the responsive group (F1-score = 0.474).

### 3.2. Predicted Responsiveness to Cognitive Training Interventions

We used the best-performing models for near-transfer effects for each type of cognitive training and applied them on the whole dataset to predict the individual responsiveness of healthy elderly participants to all cognitive training interventions considering their baseline characteristics.

**Figure 2:**
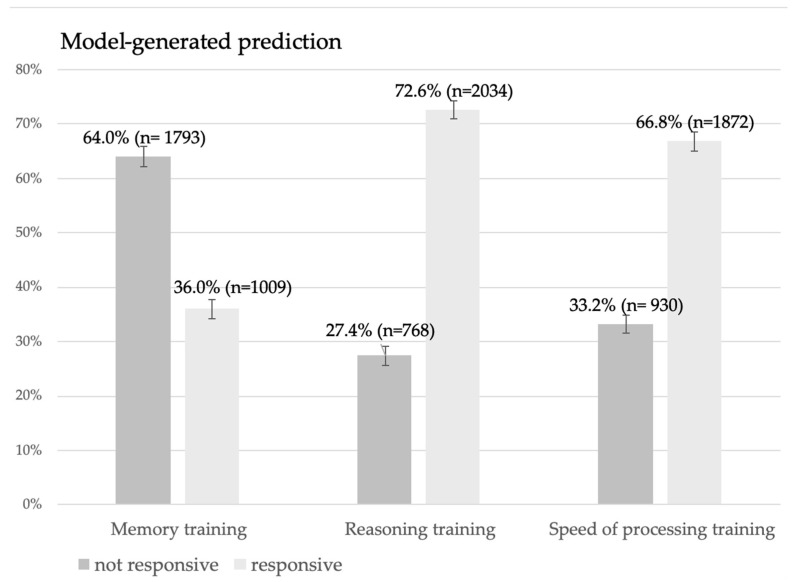
Model-generated prediction of responsiveness to all interventions on individual level.

Our study benefited from having large statistical power, attributable to the large sample size, which inflated statistical significance and enabled us to identify statistically significant results when analyzing model-predicted responsiveness to cognitive training, even when the differences were trivial. Therefore, we applied more conservative criteria for interpreting the results. Regardless of statistical significance, the results were not interpreted affirmatively if they had low effect sizes, because of limited practical significance. Accordingly, post hoc analyses interpretation also focused solely on statistically significant results but only with medium or large effect sizes. As shown in Figure 2, based on predicted values, 36% of participants are predicted responders to memory training.

For the other two interventions, it was predicted for most participants to be responders. According to the prediction, 72.6% of participants would be responders to reasoning cognitive training, and 66.8% of participants would be responders to speed-of-processing training. There was a statistically significant difference between model-generated predictions of responsiveness for different cognitive interventions (Cochran’s Q = 860.1; df = 2, *p* < 0.001; maximum-corrected η^2^ = 0.15), with post hoc analyses showing statistically significant large effect sizes between predictions of responsiveness to memory training compared to predictions for reasoning training (McNemar test Chi-square = 844.94, *p* < 0.001; g = 0.413), i.e., speed-of-processing training (McNemar test Chi-square = 481.56, *p* < 0.001; g = 0.280).

**Figure 3:**
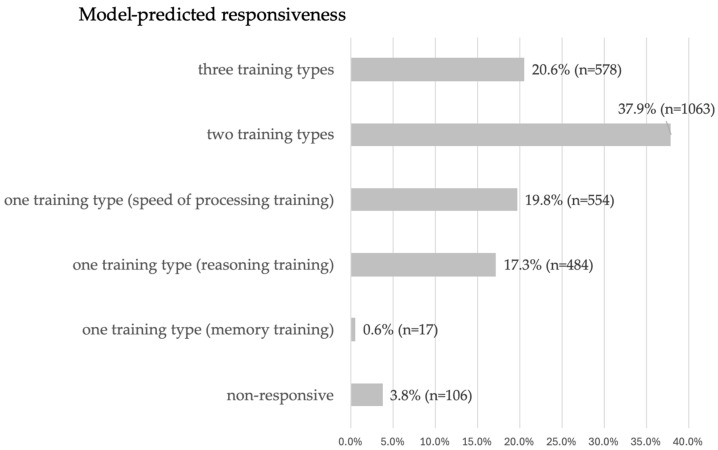
Model-predicted responsiveness categorization.

Model-predicted responsiveness to cognitive training was defined by summing the number of predicted positive near-transfer effects (based on baseline individual characteristics) of all three interventions on an individual level within six possible categories (Figure 3). Based on classification, only 3.8% of participants were predicted non-responders to any intervention, while most of the participants were predicted responders to two or three types of interventions. Among the predicted responders to just one intervention, less than one percent of participants were predicted responders to memory intervention only, while, most were predicted responders to the speed-of-processing training. The distribution of frequencies in the model-predicted responsiveness categories was statistically significant (chi-square = 1516.52; df = 5, *p* < 0.001), with the largest positive deviation observed for predicted responders to two interventions (residual = 596), and the largest negative deviation for predicted responders to memory training only (residual = −450).

**Figure 4:**
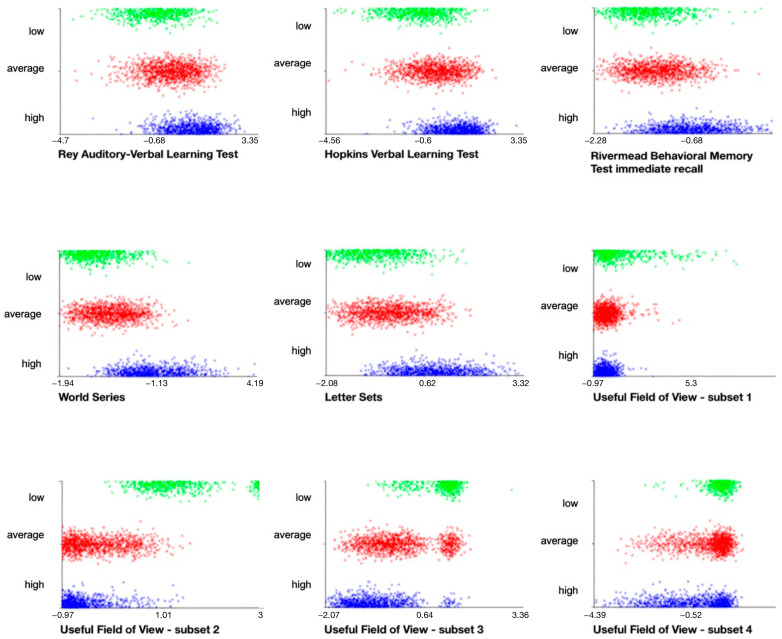
Distribution of clusters (low cognitive functioning *n* = 595, average cognitive functioning *n* = 1259 and high cognitive functioning *n* = 948) by attributes.

For exploratory purposes, K-means clustering was used to group participants based on specific cognitive functions, i.e., pre-test measures of memory, reasoning and speed of processing. The optimal number of clusters was determined by evaluating the interpretability of the solutions and analyzing the elbow plot of the within-cluster sum of squares based on the number of clusters (see Supplementary Materials for elbow curve distribution (Figure S2)). The elbow curve showed a noticeable decrease at two clusters, with an additional reduction with three cluster solution, after which curve began to plateau indicating that adding further clusters did not improve model. We identified three clusters as the optimal solution as it, compared to two-cluster solutions, robustly distinguishes high-functioning and low-functioning subjects which were our targeted groups in further analyses. Stability of the three-cluster solution was evaluated by repeating analysis across ten random initializations. Three cluster profiles were highly consistent across initializations, with similar centroids and cluster sizes indicating its robustness. Final cluster sizes and the centroid solution along with minimum and maximum values across random initializations are provided in Supplementary Materials (Table S9). Cluster 1 represents the high-cognitive-functioning group, characterized by the highest scores across all cognitive measures. Cluster 2 consists of participants with average cognitive functioning, with scores around the mean on all measures, while Cluster 3 includes the low-cognitive-functioning group, showing the lowest scores in all cognitive measures (Figure 4).

The results regarding the model-predicted responsiveness to cognitive training among participants across all examined characteristics were statistically significant. Regardless of statistical significance, the results for gender, subjective measures of everyday functioning and depressive symptoms were not further interpreted due to the low effect sizes. Main effects and post hoc analyses are presented in Table 10, Table 11, Table 12 and Table 13.

Regarding the main effects, medium effect sizes were observed for differences in age, while years of education and cognitive profile groups (derived from cluster analysis) showed near-large effect sizes. Large effect sizes were found for differences in baseline general cognitive functioning (MMSE), memory, reasoning, processing speed and objective everyday functioning.

Post hoc analyses revealed that predicted non-responders and predicted responders to only speed-of-processing training were older compared to predicted responders to reasoning training only (medium effects) and compared to predicted responders to all training types (medium effects).

Elderly participants who were predicted non-responders had less years of education compared to predicted responders to all interventions (medium effect). Predicted responders to only speed-of-processing training had less years of education compared to predicted responders to two (medium effect) or three interventions (large effect). Additionally, predicted responders to only reasoning training had less years of education compared to predicted responders to all training types (medium effect).

Predicted non-responders and predicted responders to speed-of-processing training only had lower baseline general cognitive functioning (MMSE) compared to predicted responders to reasoning training only (medium effect/large effect), or to two (medium effect/large effect) or three interventions (large effects).

Predicted non-responders had lower baseline memory compared to predicted responders to only reasoning training (large effect) or to all training interventions (large effect). Predicted responders to speed-of-processing training only had lower baseline memory compared to predicted responders to reasoning training only (large effect), or predicted responders to two (large effect) or three intervention types (large effect).

Subjects who were predicted non-responders and predicted responders to only speed-of-processing training had lower baseline reasoning compared to predicted responders to only reasoning training (medium/large effect), or to two (medium/large effect) or all three interventions (large effects).

Predicted non-responders had slower baseline speed of processing (higher result) compared to predicted responders to only reasoning training (medium effect), and had faster speed of processing (lower result) compared to predicted responders to speed-of-processing training only (large effect). Predicted responders to only reasoning training have faster speed of processing (lower result) compared to predicted responders to only speed-of-processing training (large effect) or to to two (medium effect) or three (large effect) training interventions.

Predicted non-responders had lower baseline objective everyday functioning (EPT) compared to predicted responders to only memory training (medium effect) or only reasoning training (medium effect), as well as compared to predicted responders to two (medium effect) or three types of intervention (large effect). Additionally, predicted responders to only speed-of-processing training had lower baseline EPT compared to predicted responders to only reasoning training, and predicted responders to two or three interventions (large effects). Predicted responders to reasoning training only had lower baseline EPT compared to predicted responders to all interventions (medium effect).

Elderly participants who were in cluster analysis classified as high functioning were, compared to other cognitive profiles, overrepresented in the category of predicted responders to all cognitive training interventions, and to a lesser extent, to the category of predicted responders to reasoning training or two training types, while participants classified as low cognitive functioning were overrepresented in the category of predicted responders to speed-of-processing training only. Notably, the average functioning group was overrepresented in the predicted non-responsive group. In contrast, the high-functioning group was underrepresented in the category of predicted non-responders.

## 4. Discussion

The aim of our study was to develop and test machine learning predictive models of the effects of different types of cognitive training considering baseline characteristics of healthy elderly participants and utilize those models to differentiate and analyze participants regarding their proposed responsiveness to different cognitive training interventions.

We used data from the ACTIVE study that examined the effects of cognitive training considering comparisons of intervention groups and control groups on various measures of effect, with a focus on functional outcomes of the interventions in a longitudinal perspective. In our study, the interest was not in group comparisons, but rather predictions and analyses at the individual level regarding cognitive outcomes.

The goals were motivated by the aspiration to personalize cognitive training (Shani et al., 2019) through the collection of participant characteristics important for the personalization process, in the form of applying machine learning algorithms that would enable the selection of an appropriate intervention for each individual participant.

Selected machine learning models demonstrated discrimination (AUC) above chance between responsive and non-responsive elderly individuals based on their baseline characteristics for both the near and far effects and all three cognitive interventions. In the step of feature selection, we decided to include all features in all models across all types of cognitive training, as removing features from the models did not substantially improve models.

When comparing AUC values, models for near transfer, in relation to far-transfer models, showed better discriminative ability in differentiating who was responsive to cognitive training and who was not. This result is expected since studies show higher near-transfer effects than far transfer (Basak et al., 2020; Mewborn et al., 2017) or even no far-transfer effects at all (Melby-Lervåg et al., 2016; Sala et al., 2019). Additionally, in the ACTIVE study, whose data were used for developing our models, near-transfer effects were well established and durable through five years (K. Ball et al., 2002; Willis et al., 2006), but interventions did not produce effects on other cognitive functions apart from those trained, meaning there was no far transfer. Although in the ACTIVE study, far-transfer effects did not reach statistical significance on the between-group level, there were some participants who improved with intervention in far-transfer measures and our models were able to discriminate them slightly better than by chance.

Nonetheless, compared to models for near transfer, which yielded large effect sizes for memory training and speed-of-processing training, and a small effect size for reasoning training, far transfer models demonstrated weak effects, with AUC values for reasoning training and speed-of-processing training slightly above 0.6, and for memory training below 0.6, near the boundary of a small effect size. Although AUC value labels can vary among authors as they are arbitrary and must be interpreted in a clinical context, values of 0.6 or lower are often considered failed or random (de Hond et al., 2022); therefore, caution should be taken in their interpretation and valuing their practical use.

Furthermore, inspection of model performance at the level of prediction of the responsive class showed that near-transfer models generally outperformed far-transfer models. The strongest models were the near-transfer models for reasoning training and speed-of-processing training, both of which showed relatively high recall and precision. Although the near-transfer model for memory training demonstrated comparatively high overall discrimination (AUC), it showed weaker performance in identifying responsive class. Recall was relatively high, but precision was low, resulting in moderate overall performance. Similarly, the far-transfer model in speed-of-processing training also showed relatively high recall but low precision when predicting responsive class. Nonetheless, the far-transfer models for memory and reasoning training performed poorly both regarding discriminative ability (AUC) and predicting responsive class (low recall and precision).

Considering the results of our study, we could agree with the observation of authors who have pointed out that focus should not be on unreliable far-transfer effects, rather it would be more beneficial to further seek improvements in well-established near-transfer effects, which could also offer practical benefits (Gobet & Sala, 2023).

Compared to traditional statistical methods, certain machine learning algorithms have higher complexity and can tolerate high-dimensional data with non-linear associations and interactions (Jiang et al., 2020), and therefore more complex algorithms typically result in best performance when comparing various machine learning algorithms (Orrù et al., 2020). Accordingly, in our study, more complex algorithms tended to perform the best. This suggests higher sophistication in data above simple linear relationships between individual characteristics and transfer measures. In line with this, non-linear associations were found in the cognitive training study (Feng et al., 2023) when inspecting characteristics of individuals and learning trajectories in working memory training revealing machine learning to be useful in detecting those types of relationships.

### 4.1. Predicted Responsiveness to Cognitive Training

The aim was to further inspect if we could utilize models for near-transfer effects to predict responsiveness to different types of cognitive training and find differences in older adults regarding their proposed responsiveness.

Our model-predicted findings suggest that personalization is not only desirable but necessary, given that most participants are likely to benefit only from specific types of cognitive training. Specifically, for a small subset of individuals (fewer than 5%), the model predicted to be non-responders, meaning that inclusion in any type of cognitive training would potentially not benefit them (and it might be advisable include them in other types of interventions). In contrast, about one fifth of participants were predicted responders to all types of cognitive training, implying that for them we could expect a positive near transfer regardless of the training type they received and no selection of an appropriate program would be needed. However, according to predictions, most participants would be responsive to only certain types of cognitive training, so selecting the appropriate type for each individual would be advisable in order to maximize expected efficacy. This indicates that personalizing cognitive training is worthwhile, as it increases the likelihood of achieving an effect with intervention.

When we compare the predicted responsiveness to each type of cognitive training, we can expect that the largest number of participants were predicted to respond to reasoning and speed-of-processing training, whereas a considerably smaller number of participants would be responsive to memory training only. The reported results, expectedly, are distributed similarly to those observed for the effectiveness of individual types of cognitive training in the ACTIVE study (K. Ball et al., 2002). Although all three types of training incorporated some form of individualization like providing feedback (Jobe et al., 2001), the nature and extent of this individualization appear to have differed across training types, which may underline the observed differences. It is possible that the speed-of-processing training proved to be effective for more individuals because it was the only training delivered in a computerized format with adaptive tasks, whose difficulty adjusted to the user’s task performance (Jobe et al., 2001). Adaptive tasks are generally shown to be more effective than non-adaptive ones (Pedullà et al., 2016), as are computerized versions compared to paper-and-pencil formats (Câmara et al., 2025). In contrast to memory training, the reasoning training, although non-computerized, was further individualized by offering two training levels, allowing the intervention to be adjusted to individual differences in baseline abilities (Jobe et al., 2001) which may have resulted in greater (predicted) efficiency compared to memory training.

A clear pattern of results was inspected when comparing the individual characteristics of subjects with different predicted responsiveness to cognitive training. We observed that, given the baseline cognitive profile to which the subjects belonged based on cluster analysis, high-cognitive-functioning subjects were overrepresented in the category of predicted responders to all interventions, or to a lesser extent to two interventions or to reasoning training alone, while those with a low cognitive profile were predominantly in the category of responders to speed-of-processing training only. Also, if we compare predicted non-responders, or predicted responders only to speed-of-processing training, with predicted responders to more than one intervention (or reasoning training only), we can expect that the former are on average disadvantaged, being older, less educated and having lower baseline levels on measures of cognitive functioning and objective everyday functioning.

Although our findings are model generated rather than empirical observations and therefore should be interpreted cautiously, when analyzing number of effective interventions, a pattern is in accordance with magnification effect. Specifically, considering different interventions overall, initially advantaged individuals are likely to benefit most, including a wider range of interventions, whereas those with an initial disadvantage are predicted to benefit only from specific interventions (typically speed-of-processing training) or may be non-responsive to any intervention. However, when we classified individuals according to cognitive profile, predicted non-responders were overrepresented in the average cognitive functioning group, whereas in the low-cognitive-functioning-group, the category of predicted responders to speed-of-processing training only was overrepresented. Furthermore, considering an overall small proportion of predicted non-responders, it is important to highlight that individuals who were initially disadvantaged would, based on predictions, in most cases, benefit from certain cognitive training.

Out of individual interventions, for those who were initially advantaged, reasoning training appears to be the most appropriate. In contrast, for those who are initially disadvantaged, speed-of-processing training is predicted to be the most suitable. Reasoning training in the ACTIVE study was strategy based and more demanding, therefore needed individualization (Jobe et al., 2001). The acquisition of strategies requires efficient existing cognitive resources (Shaw & Hosseini, 2021), which is why strategic training typically results in magnification effects, a finding also aligning with our results regarding reasoning training. Speed-of-processing training emerges as the main intervention suitable for individuals who exhibited lower baseline levels of the assessed characteristics. Speed-of-processing training used in the ACTIVE study was process based, and this type of intervention also appears to be effective for participants with initially lower functioning, as supported by previous findings leaning toward compensation effects regarding this type of training (Shaw & Hosseini, 2021). Existing studies also indicate that speed-of-processing training is among the most effective single-domain intervention (Basak et al., 2020) and, as shown in the ACTIVE study, is suitable for individuals with a wide range of characteristics (K. K. Ball et al., 2013).

### 4.2. Limitations and Future Directions

When analyzing the performance of machine learning models, it can be observed that class imbalance influenced its performance by favoring the majority class. Thus, in the reasoning and speed-of-processing training models for near-transfer effects, where the responsive class was predominant, both recall and precision were relatively high. Conversely, although the memory training model showed better overall discrimination (AUC) compared to the reasoning training model, it performed less well in predicting the responsive (minority) class, exhibiting a moderate balance between recall and precision.

In our research, the focus was on subjects who were responsive to the intervention, therefore we classified participants with no significant effect and negative effect into one heterogeneous group. This allowed us to improve sensitivity, i.e., recall of the model, that is, we enabled the model to more easily recognize those who were truly responsive to cognitive training. On the other hand, among those who were classified as not responsive were those who achieved a slight positive effect. These responders were perhaps more similar to those who achieved a significant effect than to those who had a negative effect. This can lead to the model categorizing those who we classified as truly non-responsive into responsive, resulting in a higher number of false positives, thereby reducing the specificity of the model. The effect of false positives was evident in some of our models where responsive class was the minority, where despite relatively high recall, precision in predicting responsive class was reduced due to the large number of false-positive classifications. However, when it comes to cognitive training, which could benefit a person without indications of harm, we do not want to miss potential users, so it is better for the model to give false-positive results than, conversely, to miss users who would benefit from the intervention.

The goal of the study was to enable selection of the appropriate intervention for a specific individual when applying cognitive training to increase the likelihood of a positive effect of cognitive training. Our study was in the feasibility phase, that is, the stage of testing the viability of a given paradigm or project (Green et al., 2019), and it remains to be further examined whether the models would function effectively in practice.

To truly test if the models could generalize outside of the sample from the ACTIVE study, they should be externally validated on another sample (Jiang et al., 2020). A comparison of model performance on a new sample of participants with the performance observed in our study is needed to assess if the models are valid for the target population on which we intend to apply the intervention.

The advantage is that the ACTIVE study used standardized measures and tests, which could be used when replicating the research and conducting external validation of the models, which could facilitate the model generalization.

Given the pilot nature of this work, the findings would additionally benefit from external validation in more diverse samples, including clinical populations such as older adults with cognitive impairment (e.g., mild cognitive impairment or dementia).

To apply the created prediction models in a real-word setting and also test their practical usability, the first step is to collect the subject’s sociodemographic data and conduct initial testing to examine cognitive and daily functioning, as well as depressive symptoms—that is, to collect the data which were features in the machine learning models. After data collection, we could apply those models on collected baseline data of an individual to predict whether they would respond to a certain cognitive training intervention or not. A subject could be included in certain intervention(s) if the model predicts their responsiveness to that type of training based on their baseline characteristics. After completion of the selected cognitive training program, there is also a need for evaluation of efficacy of applied intervention to inspect whether our prediction was accurate to provide information about the practical relevance of our selection method.

The criterion for inclusion in cognitive training would be the predicted responsiveness, which is based on a binary responsive/non-responsive outcome derived from the RCI in our study. The RCI is a measure of statistically reliable change, which indicates that the observed change is not a result of measurement error, i.e., fluctuations due to instrument unreliability. However, the RCI alone does not indicate whether the change is clinically significant. To claim a clinically significant change, it is necessary, in addition to reliable change, to assess the functional status of the participant by inspecting whether the outcome measure post intervention falls within the range of the functional population (Jacobson & Truax, 1991). In the ACTIVE study, the sample consisted of cognitively healthy adults and therefore no additional criterion of clinically significant change was applied (although some participants could be classified as having mild cognitive impairment). If the current models were to be applied to a clinical population with cognitive impairment and functional deficits, it would be advisable to include an additional criterion when measuring intervention effectiveness. Specifically, it would be advisable to inspect whether subjects improve on cognitive tests to a level within the range of normal cognitive functioning or whether there is functional improvement, for example, in daily life (e.g., in objective measures of everyday functioning or functioning reported by a caregiver).

When selecting the intervention considering model predictions based on individual characteristics, in addition to proposing a single intervention that could be applied, there is the possibility of inclusion in multidomain cognitive training, which may yield greater efficacy (Basak et al., 2020).

Cognitive training is an intervention that requires considerable mental effort, and for it to be effective, individuals need to participate for an extended period (Alloway et al., 2013; Stepankova et al., 2014). This can discourage some participants, leading them to give up from the intervention. If, additionally, predictions indicate that training is unlikely to be effective, this may further reduce motivation and even impact efficacy of the cognitive training (Jaeggi et al., 2014). On the other hand, if potential users were encouraged to participate in cognitive training based on an initial assessment suggesting a higher likelihood of benefit, this could have a motivating effect and potentially reduce dropout rates. If it were predicted that cognitive training is not suitable for a particular individual, they could be offered and directed toward other interventions that might be more suitable (e.g., working memory or executive function training).

Machine learning models such as those developed in our study have potential to be implemented into adaptive cognitive training platforms. In doing so, consideration should not be limited to individual characteristics examined in the present study but should also include other individual factors that may impact training outcomes, such as adherence to the training, motivation or health-related difficulties that could potentially interfere with the participation. Adaptive or hybrid protocols could additionally be adjusted regarding task difficulty, content or duration of the intervention. This would allow training protocols to be tailored to individual profiles, thereby supporting more targeted and efficient cognitive interventions.

Although decision making based on machine learning models has the potential to be more objective than ad hoc decisions of clinicians, it raises some ethical considerations regarding potential biases of those models, transparency of their decisions or questions of responsibility. Therefore, ethical aspects should be well established before their implementation and decision making should be supervised by trained professionals, especially when, as in our case, models are not completely accurate.

Even though our models performed above the chance level, the results–particularly for the far-transfer models–remain limited in predictive accuracy and unreliable. The results suggest that far-transfer prediction remains a methodological challenge, and that at this stage, the current models should be viewed as exploratory rather than clinically applicable.

In contrast to more exact scientific domains, predicting human behavior involves multiple interacting factors that are only partially observable through testing and questionnaire-based measures. Consequently, lower predictive performance can be expected. Accordingly, our model performances are comparable to those of similar studies on cognitive training.

For example, Vladisauskas et al. (2022) applied several machine learning algorithms to predict effects of executive function training based on pre-existing individual cognitive differences in children. In their study, the Support Vector classifier performed best with average accuracy = 0.67 (AUC = 0.707). Furthermore, Feng et al. (2023) developed a binary tree classification model aimed at predicting individuals’ learning patterns in cognitive training based on a range of individual differences (cognitive abilities, personality traits, motivational factors, video game experience, health status, bilingualism and socioeconomic status). On a holdout test set, the first-stage model achieved an accuracy of 0.74 in identifying high performers, while the second-stage model reached an accuracy of 0.59 in differentiating between lower-performer groups, with an overall classification accuracy of around 0.51 across learning patterns (over chance level 33%).

Although the ACTIVE study is one of the largest clinical studies of cognitive training, it is possible that the suboptimal results are due to the relatively small dataset, as machine learning methods are typically applied to larger datasets, and it has generally been shown that predictive performance increases with sample size (Jiang et al., 2020). When building predictive models, we try to avoid overfitting the data by cross-validating the model in the training stage and testing it in the hold-out sample to improve the generalization of models outside of the sample, potentially at the cost of lower performance of the models. On the other hand, increasing the complexity of the model can enhance overfitting. Specifically, we included a relatively large number of features in our models, and although some machine learning algorithms can better tolerate a high predictor-to-observation ratio compared to classical statistical models (Cortes & Vapnik, 1995), increasing the number of input variables nevertheless raises the likelihood of overfitting (Coutanche & Hallion, 2020). Choosing the best-performing models from numerous algorithms and hyperparameter configurations may also limit the replicability of results in external samples. These potential threats to model stability and replicability highlight the need for prospective validation designs which would allow model evaluation under real-world conditions in order to draw more reliable conclusions. This is further amplified by the fact that models were developed using data from the ACTIVE study sample, which is geographically and culturally limited (U.S. population); therefore, their generalizability beyond the original sample characteristics remains uncertain. Additionally, the sample was relatively dated, and in comparison, contemporary older adults may be more digitally literate, more highly educated and exposed to improved health care and longevity which could improve their cognitive performance, especially on computerized cognitive tasks and interventions which may further limit generalizability.

## 5. Conclusions

Our research provides a novel direction for the further development of the field of cognitive training by highlighting the potential of machine learning methods for the personalization of interventions and their practical implementation. We found a better than chance discrimination (AUC range from 0.56–0.74) of selected machine learning algorithms when predicting near- and far-transfer effects of different cognitive training programs, based on sociodemographic characteristics, measures of cognitive and everyday functioning, and depressive symptoms, although models for far-transfer outcomes remain unreliable and unsuitable for clinical decision making at this stage. Furthermore, when applying near-transfer models to predict responsiveness to cognitive training, we demonstrated that, depending on baseline characteristics, healthy older adults would respond differently to various cognitive training interventions, thereby highlighting the usefulness of a personalized cognitive training approach. Regarding the impact of baseline characteristics, our findings suggest that based on model predictions, initially advantaged individuals would benefit from a wider range of interventions compared to those initially disadvantaged, supporting the magnification effect. As a proof-of-concept study, our findings require external validation and real-time adaptive trials to confirm their practical utility, including clinical populations such as older adults with cognitive impairment. Future research should focus on the integration of these models into adaptive cognitive training platforms which could enhance training efficacy and enable individualized, and potentially more objective, intervention decisions.

## Figures and Tables

**Table 1:** Hyperparameters tuned by algorithm.

Multilayer Perceptron
Hidden layers: a ((attribute + classes)/2), i (attributes), o (classes), t (attribute + classes) Learning Rate: 0.1, 0.3, 0.5 Momentum: 0.1, 0.2, 0.5 Training Time: 500, 1000, 1500
Support Vector Machines
The complexity parameter c= 0.1, 1, 10 Epsilon (ε): 1.0^−12^, 0.001, 0.1 Kernel: Poly Kernel, RBF Kernel
Random Forest
Depth = 0, 1, 5, 10 Number of Iterations = 100, 500, 1000, 1500, 2000

**Table 2:** Machine learning methodology workflow.

Step	Input Data	Action
1	Memory CT group	Build ML models for memory intervention (near- and far-transfer)
	Reasoning CT group	Build ML models for reasoning intervention (near- and far-transfer)
	Speed-of-processing CT group	Build ML models for speed of processing intervention (near- and far-transfer)
2	Whole dataset (all groups combined, including control group)	Apply selected ML models for all intervention types (near-transfer)
3	Predicted outcomes of all three models	Define and analyze predicted responsiveness to cognitive training

**Table 3:** Comparison of sociodemographic characteristics regarding in/exclusion criteria (missing values) and total sample by intervention group.

	Memory Training			Reasoning Training			Speed-of-Processing Training		
	Excluded (*n* = 62)	Included (*n* = 641)	Total (*n* = 703)	Excluded (*n* = 70)	Included (*n* = 629)	Total (*n* = 699)	Excluded (*n* = 49)	Included (*n* = 653)	Total (*n* = 702)
Age; M (SD)	74.95 (6.44)	73.39 (5.97)	73.53 (6.02)	74.33 (6.63)	73.45 (5.65)	73.53 (5.76)	73.73 (6.99)	73.40 (5.68)	73.42 (5.78)
Years of education; M (SD)	13.24 (2.84)	13.62 (2.72)	13.59 (2.73)	13.44 (2.88)	13.51 (2.67)	13.50 (2.69)	13.31 (2.79)	13.68 (2.68)	13.65 (2.68)
Gender, female; *n* (%)	45 (72.6)	492 (76.8)	537 (76.4)	57 (81.4)	480 (76.3)	537 (76.8)	41 (83.7)	497 (76.1)	538 (76.6)

**Table 4:** Descriptive statistic of included features in models by intervention group.

Feature		Memory Training *n* = 641	Reasoning Training *n* = 629	Speed-of-Processing Training *n* = 653
Age	M (SD); min–max	73.39 (5.97); 65–93	73.45 (5.65); 65–90	73.40 (5.68); 65–91
Years of Education	M (SD); min–max	13.62 (2.72); 5–20	13.51 (2.67); 4–20	13.68 (2.68); 5–20
Gender, female	N (%)	492 (76.76%)	480 (76.31%)	497 (76.11%)
MMSE total	M (SD); min–max	27.39 (2.01); 23–30	27.31 (1.98); 23–30	27.45 (1.97); 23–30
HVLT	M (SD); min–max	26.28 (5.33); 4–36	25.89 (5.40); 4–36	26.19 (5.31); 6–36
AVLT	M (SD); min–max	48.93 (10.04); 8–71	48.64 (9.79); 17–70	48.36 (10.29); 0–72
Rivermead	M (SD); min–max	6.32 (2.82); 0–15.5	6.38 (2.78); 0–17	6.28 (2.75); 0–15.5
Word Series	M (SD); min–max	9.86 (4.95); 0–25	9.51 (4.91); 0–28	9.66 (4.83); 0–30
Letter Sets	M (SD); min–max	5.98 (2.75); 0–14	5.85 (2.79); 0–13	5.76 (2.64); 0–14
UFOV Task 1 score	M (SD); min–max	30.70 (40.38); 16–500	29.00 (32.27); 16–250	29.79 (37.63); 16–343
UFOV Task 2 score	M (SD); min–max	127.69 (123.29); 16–500	125.48 (118.99); 16–500	129.93 (117.62); 16–500
UFOV Task 3 score	M (SD); min–max	312.76 (134.56); 63–500	316.95 (136.31); 63–500	322.07 (131.61); 66–500
UFOV Task 4 score	M (SD); min–max	452.42 (71.02); 173–500	457.32 (67.90); 153–500	455.34 (71.55); 170–500
IADL performance	M (SD); min–max	4.56 (5.13); 0–26	4.37 (5.01); 0–23	4.22 (4.92); 0–25
IADL difficulty	M (SD); min–max	1.32 (2.29); 0–17	1.44 (2.48); 0–20	1.45 (2.47); 0–16
ADL performance	M (SD); min–max	0.30 (0.86); 0–10	0.33 (0.90); 0–8	0.26 (0.90); 0–9
EPT	M (SD); min–max	19.08 (5.64); 2–28	18.75 (5.69); 0–28	18.97 (5.64); 2–28
CES-D	M (SD); min–max	5.19 (4.98); 0–27	5.40 (5.42); 0–34	5.08 (4.88); 0–27

**Table 5:** The best-performing predictive machine learning models for the memory training regarding near and far transfer (weighted average model performance).

Outcome	Algorithm	True-Positive Rate/Recall [95% CI]	False-Positive Rate [95% CI]	Precision [95% CI]	F1-Score [95% CI]	Brier Score [95% CI]	AUC [95% CI]
			Training with 10-k CV (80% dataset)				
near transfer	Ensemble *	0.691 [0.652, 0.732]	0.345 [0.283, 0.407]	0.742 [0.707, 0.776]	0.707 [0.680, 0.735]	0.201 [0.176, 0.225]	0.706 [0.651, 0.761]
			Test (20% dataset)				
		0.667 [0.586, 0.748]	0.314 [0.234, 0.394]	0.783 [0.712, 0.854]	0.698 [0.635, 0.761]	0.213 [0.161, 0.264]	0.727 [0.598, 0.856]
			Training with 10-k CV (80% dataset)				
far transfer	Naïve Bayes	0.611 [0.569, 0.653]	0.488 [0.445, 0.531]	0.592 [0.550, 0.635]	0.591 [0.552, 0.630]	0.261 [0.229, 0.293]	0.569 [0.519, 0.619]
			Test (20% dataset)				
		0.574 [0.489, 0.659]	0.600 [0.516, 0.684]	0.548 [0.462, 0.634]	0.559 [0.482, 0.637]	0.263 [0.187, 0.339]	0.554 [0.450, 0.658]

* Ensemble method combining five classifiers: Naïve Bayes, Logistic Regression, Multilayer Perceptron (hidden layers = i, learning rate = 0.3, momentum = 0.2, training time = 500), Support Vector Machines (c = 10, ε = 1.0^−12^, RBFkernel), Random Forest (depth = 10, iteration = 500).

**Table 6:** Summary of model’s performance and confusion matrix of selected near-transfer models.

Near-Transfer Models	Confusion Matrix			Overall Model	Model Performance for Responsive Class (1) [95% CI]			
Memory Training	*n* (% Row, % Column)			Accuracy	True-Positive Rate/Recall	**Precision**	**F1-Score**	**AUC**
	Predicted							
Actual	Not responsive	Responsive	Total	0.691	0.692	0.340	0.456	0.727
Not responsive	68 (66.0%, 89.5%)	35 (34.0%, 66.0%)	103 (79.8%)	[0.652, 0.732]	[0.515–0.869]	[0.212–0.467]	[0.335, 0.577]	[0.598, 0.856]
Responsive		8 (30.8%, 10.5%)	18 (69.2%, 34.0%)	26 (20.2%)				
Total		76 (58.9%)	53 (41.1%)	129 (100%)				
Reasoning training	Predicted							
Actual	Not responsive	Responsive	Total	0.667	0.763	0.726	0.744	0.631
Not responsive	23 (50.0%, 54.8%)	23 (50.0%, 27.4%)	46 (36.5%)	[0.585, 0.749]	[0.670–0.856]	[0.631–0.821]	[0.677, 0.811]	[0.533, 0.729]
Responsive	19 (23.8%, 45.2%)	61 (76.3%, 72.6%)	80 (63.5%)					
Total	42 (33.3%)	84 (66.7%)	126 (100%)					
Speed of processing training	Predicted							
Actual	Not responsive	Responsive	Total	0.705	0.767	0.786	0.776	0.742
Not responsive	25 (58.1%, 55.6%)	18 (41.9%, 21.4%)	43 (33.3%)	[0.627, 0.784]	[0.678–0.856]	[0.698–0.873]	[0.714, 0.838]	[0.652, 0.832]
Responsive		20 (23.3%, 44.4%)	66 (76.7%, 78.6%)	86 (66.7%)				
Total		45 (34.9%)	84 (65.1%)	129 (100%)				

**Table 7:** Summary of model’s performance and confusion matrix of selected far-transfer models.

Far-Transfer Models	Confusion Matrix			Overall Model	Model Performance for Responsive Class (1) [95% CI]			
Memory Training	*n* (% Row, % Column)			Accuracy	True-Positive Rate/Recall	Precision	F1-Score	AUC
	Predicted							
Actual	Not responsive	Responsive	Total	0.574	0.238	0.303	0.267	0.554
Not responsive	64 (73.6%, 66.7%)	23 (26.4%, 69.7%)	87 (67.4%)	[0.489, 0.659]	[0.109, 0.367]	[0.146–0.460]	[0.118–0.417]	[0.450, 0.658]
Responsive	32 (76.2%, 33.3%)	10 (23.8%, 30.3%)	42 (32.6%)					
Total	96 (74.4%)	33 (25.6%)	129 (100%)					
Reasoning training	Predicted							
Actual	Not responsive	Responsive	Total	0.635	0.400	0.421	0.410	0.617
Not responsive	64 (74.4%, 72.7%)	22 (25.6%, 57.9%)	86 (68.3%)	[0.551, 0.719]	[0.248–0.552]	[0.264–0.578]	[0.302, 0.520]	[0.509–0.725]
Responsive	24 (60.0%, 27.3%)	16 (40.0%, 42.1%)	40 (31.7%)					
Total	88 (69.8%)	38 (30.2%)	126 (100%)					
Speed of processing	Predicted							
Actual	Not responsive	Responsive	Total	0.542	0.675	0.365	0.474	0.603
Not responsive	44 (48.4%, 77.2%)	47 (51.6%, 63.5%)	91 (69.5%)	[0.457, 0.627]	[0.530–0.820]	[0.255–0.475]	[0.375, 0.573]	[0.495–0.711]
Responsive	13 (32.5%, 22.8%)	27 (67.5%, 36.5%)	40 (30.5%)					
Total	57 (43.5%)	74 (56.5%)	131 (100%)					

**Table 8:** The best-performing predictive machine learning models for the reasoning cognitive training regarding near and far transfer (weighted average model performance).

Outcome	Algorithm	True-Positive Rate/Recall [95% CI]	False-Positive Rate [95% CI]	Precision [95% CI]	F1-Score [95% CI]	Brier Score [95% CI]	AUC [95% CI]
			Training with 10-k CV (80% dataset)				
Near transfer	Support Vector Machines *	0.650 [0.608, 0.692]	0.443 [0.400, 0.486]	0.640 [0.598, 0.682]	0.629 [0.583, 0.675]	0.350 [0.308, 0.392]	0.604 [0.555, 0.654]
			Test (20% dataset)				
		0.667 [0.585, 0.749]	0.404 [0.318, 0.490]	0.661 [0.578, 0.744]	0.663 [0.605, 0.721]	0.333 [0.251, 0.416]	0.631 [0.533, 0.729]
			Training with 10-k CV (80% dataset)				
Far transfer	Random Forest **	0.708 [0.668, 0.748]	0.435 [0.392, 0.478]	0.710 [0.670, 0.750]	0.709 [0.675, 0.743]	0.212 [0.176, 0.248]	0.672 [0.622, 0.722]
			Test (20% dataset)				
		0.635 [0.551, 0.719]	0.491 [0.404, 0.578]	0.630 [0.546, 0.714]	0.632 [0.572, 0.692]	0.223 [0.151, 0.296]	0.617 [0.509–0.725]

* c = 1, ε = 10^−12^, RBFkernel; ** depth = 5, iteration = 500.

**Table 9:** The best-performing predictive machine learning models for the speed-of-processing cognitive training regarding near and far transfer (weighted average model performance).

Outcome	Algorithm	True-Positive Rate/Recall [95% CI]	False-Positive Rate [95% CI]	Precision [95% CI]	F1-Score [95% CI]	Brier Score [95% CI]	AUC [95% CI]
			Training with 10-k CV (80% dataset)				
Near transfer	Random Forest *	0.770 [0.734, 0.806]	0.305 [0.265, 0.345]	0.768 [0.732, 0.804]	0.769 [0.743, 0.795]	0.162 [0.130, 0.194]	0.821 [0.786, 0.856]
			Test (20% dataset)				
		0.705 [0.627, 0.784]	0.357 [0.274, 0.440]	0.709 [0.631, 0.787]	0.707 [0.651, 0.763]	0.199 [0.130, 0.268]	0.742 [0.652, 0.832]
			Training with 10-k CV (80% dataset)				
Far transfer	Logistic Regression	0.600 [0.558, 0.642]	0.401 [0.359, 0.443]	0.673 [0.633, 0.713]	0.619 [0.589, 0.649]	0.239 [0.203, 0.276]	0.619 [0.564, 0.674]
			Test (20% dataset)				
		0.542 [0.457, 0.627]	0.383 [0.300, 0.466]	0.648 [0.566, 0.730]	0.558 [0.497, 0.619]	0.244 [0.170, 0.317]	0.603 [0.495, 0.711]

* depth = 10, iteration = 500.

**Table 10:** Model-predicted responsiveness to cognitive training (CT) interventions by baseline sociodemographic characteristics.

Predicted Responsiveness		Non-Responsive *n* = 106 (0)	Memory CT Only *n* = 17 (1)	Reasoning CT Only *n* = 484 (2)	Speed CT Only *n* = 554 (3)	Two CTs *n* = 1063 (4)	All CTs *n* = 578 (5)
Age	Median (IQR)	79 (73–82)	78 (76–79)	72 (68–76)	77 (71–82)	72 (69–77)	71 (68–74)
	[min, max]	[65, 93]	[68, 86]	[65, 90]	[65, 93]	[65, 94]	[65, 87]
Kruskal–Wallis test, H = 276.53; *p* < 0.001, η^2^ = 0.099 (medium effect)							
Post hoc Mann–Whitney test, *p* < 0.001, r ≥ 0.3: 0 > 2 (r = 0.31); 0 > 5 (r = 0.36), 2 < 3 (r = 0.31); 3 > 5 (r = 0.41)							
Gender; Male	*n* (% row, % column)	22 (3.3; 20.8)	5 (0.7; 29.4)	141 (20.9; 29.1)	120 (17.8; 21.7)	236 (34.9; 22.2)	152 (22.5; 26.3)
Female	*n* (% row, % column)	84 (4.0; 79.2)	12 (0.6; 70.6)	343 (16.1; 70.9)	434 (20.4; 78.3)	827 (38.9; 77.8)	426 (20.0; 73.7)
Chi-square = 13.02, df = 5, *p* = 0.023, Cramer’s V = 0.068							
Years of Education	Median (IQR)	12 (11–13)	13 (13–13)	13 (12–14)	12 (11–13)	13 (12–16)	14 (13–17)
	[min, max]	[6, 20]	[10, 18]	[6, 20]	[4, 20]	[6, 20]	[9, 20]
Kruskal–Wallis test, H = 391.07; *p* < 0.001, η^2^ = 0.139 (near-large effect)							
Post hoc Mann–Whitney test, *p* < 0.001, r ≥ 0.3: 0 < 5 (r = 0.38), 2 < 5 (r = 0.31); 3 < 4 (r = 0.35); 3 < 5 (r = 0.51)							

H = Kruskal–Wallis test statistic; *p* = significance level; η^2^ = eta squared; effect size measure; df = degrees of freedom, r = effect size measure.

**Table 11:** Model-predicted responsiveness to cognitive training (CT) interventions by baseline measures of cognitive functioning.

Predicted Responsiveness		Non-Responsive *n* = 106 (0)	Memory CT Only *n* = 17 (1)	Reasoning CT Only *n* = 484 (2)	Speed CT Only *n* = 554 (3)	Two CTs *n* = 1063 (4)	All CTs *n* = 578 (5)
MMSE total	Median (IQR)	25 (23–26)	27 (25–27)	28 (27–29)	25 (24–27)	28 (27–29)	29 (27–29)
	[min, max]	[23, 29]	[24, 30]	[23, 30]	[23, 30]	[23, 30]	[23, 30]
Kruskal–Wallis test, H = 760.66; *p* < 0.001, η^2^ = 0.271 (large effect)							
Post hoc Mann–Whitney test, *p* < 0.001, r ≥ 0.3: 0 < 2 (r = 0.47); 0 < 4 (r = 0.37); 0 < 5 (r = 0.52); 2 > 3 (r = 0.54); 3 < 4 (r = 0.54); 3 < 5 (r = 0.66)							
Memory (mean z-score)	Median (IQR)	−1.03 (−1.37–(−0.58))	−1.37 (−1.75–(−0.89))	0.32 (−0.22–0.87)	−0.81 (−1.22–(−0.35))	0.24 (−0.25–0.79)	0.17 (−0.25–0.59)
	[min, max]	[−3.16, 0.29]	[−2.28, −0.32]	[−1.55, 3.35]	[−2.78, 0.97]	[−3.42, 2.53]	[−2.21, 1.90]
Kruskal–Wallis test, H= 868.40; *p* <0.001, η^2^ = 0.310 (large effect)							
Post hoc Mann–Whitney test, *p* < 0.001, r ≥ 0.3: 0 < 2 (r = 0.57); 0 < 4 (r = 0.40); 0 < 5 (r = 0.54); 2 > 3 (r = 0.65); 3 < 4 (r = 0.58); 3 < 5 (r = 0.64)							
Reasoning (mean z-score)	Median (IQR)	−0.87 (−1.13–(−0.49))	−0.39 (−0.64–(−0.25))	0.01 (−0.52–0.63)	−0.94 (−1.21–(−0.63))	0.07 (−0.37–0.62)	0.53 (0.04–1.05)
	[min, max]	[−1.68, 0.54]	[−1.24, 0.34]	[−1.58, 3.58]	[−1.88, 0.92]	[−1.58, 3.16]	[−1.07, 3.22]
Kruskal–Wallis test, H = 1082.49; *p* < 0.001, η^2^ = 0.386 (large effect)							
Post hoc Mann–Whitney test, *p* < 0.001, r ≥ 0.3: 0 < 2 (r = 0.43); 0 < 4 (r = 0.36); 0 < 5 (r = 0.56); 2 > 3 (r = 0.61); 3 < 4 (r = 0.65); 3 < 5 (r = 0.80)							
Speed of processing (mean z-score)	Median (IQR)	−0.08 (−0.24–0.12)	−0.04 (−1.97–0.07)	−0.63 (−0.90–(−0.29))	0.73 (0.45–1.20)	−0.13 (−0.54–0.31)	−0.18 (−0.38–0.20)
	[min, max]	[−1.03, 1.21]	[−0.62, 0.71]	[−1.74, 0.67]	[−0.78, 4.14]	[−1.67, 4.14]	[−1.09, 1.40]
Kruskal–Wallis test, H = 1138.13; *p* < 0.001, η^2^ = 0.408 (large effect)							
Post hoc Mann–Whitney test, *p* < 0.001, r ≥ 0.3: 0 > 2 (r = 0.44); 0 < 3 (r = 0.51); 2 < 3 (r = 0.83); 2 < 4 (r = 0.38); 2 < 5 (r = 0.53); 3 > 4 (r = 0.61), 3 > 5 (r = 0.71)							

H = Kruskal–Wallis test statistic; *p* = significance level; η^2^ = eta squared; effect size measure; df = degrees of freedom, r = effect size measure.

**Table 12:** Model-predicted responsiveness to cognitive training (CT) interventions by cognitive functioning clusters.

Predicted Responsiveness		Non-Responsive *n* = 106	Memory CT Only *n* = 17	Reasoning CT Only *n* = 484	Speed CT Only *n* = 554	Two CTs *n* = 1063	All CTs *n* = 578
Cognitive profile; high functioning *n* = 948	*n* (% row, % column)	0 a (0.0; 0.0)	0 a (0.0; 0.0)	234 b (24.7; 48.3)	0 a (0.0; 0.0)	427 c (45.0; 40.2)	287 b (30.3; 49.7)
	adjusted residual	−7.5	−3.0	7.4	−18.8	5.5	9.0
Average functioning *n* = 1259	*n* (% row, % column)	90 a (7.1; 84.9)	14 a,b (1.1; 82.4)	248 b,c (19.7; 51.2)	152 d (12.1; 27.4)	495 b,c (39.3; 46.6)	260 c (20.7; 45.0)
	adjusted residual	8.4	3.1	3.1	−9.2	1.4	0.0
Low functioning *n* = 595	*n* (% row, % column)	16 a (2.7; 15.1)	3 a,b (0.5; 17.6)	2 c (0.3; 0.4)	402 d (67.6; 72.6)	141 a (23.7; 13.3)	31 b (5.2; 5.4)
	adjusted residual	−1.6	−0.4	−12.3	33.0	−8.1	−10.5
	Chi-square = 1288.73, df = 10, *p* < 0.001, Cramer’s V = 0.480 (near-large effect)						

*p* = significance level; df = degrees of freedom. Each letter (a–d) denotes a subset of predicted responsiveness categories whose column proportions do not differ significantly from each other.

**Table 13:** Predicted responsiveness to cognitive training (CT) interventions by measures of everyday functioning.

Predicted Responsiveness		Not Responsive *n* = 106 (0)	Memory CT Only *n* = 17 (1)	Reasoning CT Only *n* = 484 (2)	Speed CT Only *n* = 554 (3)	Two CTs *n* = 1063 (4)	All CTs *n* = 578 (5)
IADL performance	Median (IQR)	3 (1–6)	7 (1–11)	3 (0–6)	3 (0–7)	3 (0–6)	3 (0–7)
	[min, max]	[0, 23]	[0, 20]	[0, 22]	[0, 25]	[0, 26]	[0, 23]
Kruskal–Wallis test, H = 16.85; *p* = 0.005, η^2^ = 0.006							
IADL difficulty	Median (IQR)	1 (0–4)	3 (1–6)	0 (0–2)	1 (0–3)	0 (0–2)	0 (0–1)
	[min, max]	[0, 16]	[0, 11]	[0, 10]	[0, 16]	[0, 20]	[0, 17]
Kruskal–Wallis test, H = 96.78; *p* < 0.001, η^2^ = 0.035							
ADL performance	Median (IQR)	0 (0–0)	0 (0–0)	0 (0–0)	0 (0–0)	0 (0–0)	0 (0–0)
	[min, max]	[0, 9]	[0, 2]	[0, 7]	[0, 11]	[0, 5]	[0, 7]
Kruskal–Wallis test, H = 46.06; *p* < 0.001, η^2^ = 0.016							
EPT	Median (IQR)	13 (9–16)	18 (16–21)	20 (16–23)	12 (9–16)	21 (17–24)	23 (20–25)
	[min, max]	[0, 22]	[13, 25]	[4, 28]	[0, 25]	[3, 28]	[10, 28]
Kruskal–Wallis test, H= 1085.92; *p* < 0.001, η^2^ = 0.389							
Post hoc Mann–Whitney test, *p* < 0.001, r ≥ 0.3: 0 < 1 (r = 0.42); 0 < 2 (r = 0.48); 0 < 4 (r = 0.39); 0 < 5 (r = 0.58); 2 > 3 (r = 0.63); 2 < 5 (r = 0.31); 3 < 4 (r = 0.65); 3 < 5 (0.79)							
CES-D	Median (IQR)	4 (1–7)	12 (6–14)	3 (1–6)	4 (1–8)	4 (1–8)	5 (2–9)
	[min, max]	[0, 25]	[0, 34]	[0, 23]	[0, 34]	[0, 28]	[0, 27]
Kruskal–Wallis test, H = 60.39; *p* < 0.001, η^2^ = 0.022							

H = Kruskal–Wallis test statistic; *p* = significance level; η^2^ = eta squared; effect size measure; r = effect size measure near- and far-transfer predictions.

## Data Availability

The original data presented in the study are openly available in https://www.icpsr.umich.edu/web/NACDA/studies/36036, https://doi.org/10.3886/ICPSR38821.v1. The data was accessed on date: 3 November 2023. The data analyses supporting the conclusions of this article are available from the authors upon reasonable request.

## Note

1 Datasets for near transfer and far transfer in speed-of-processing training were not reported separately due to negligible differences attributable to small difference in sample (10 cases). Dataset with N = 653 was used in descriptive analyses.

## Supplementary Materials

The following supporting information can be downloaded at https://www.mdpi.com/article/10.3390/jintelligence14040056/s1, Figure S1: Flowchart of machine learning methodology; Figure S2: Elbow curve; Table S1: RCI calculation results; Table S2: Correlation matrix of features; Table S3: Ranking of the feature importance by near transfer/memory training using the Information Gain Attribute Evaluator with the Ranker method, and results of the feature ablation process within 10-CV on the train dataset for the selected model; Table S4: Ranking of the feature importance by far transfer/memory training using the Information Gain Attribute Evaluator with the Ranker method, and results of the feature ablation process within 10-CV on the train dataset for the selected model; Table S5: Ranking of the feature importance by near transfer/reasoning training using the Information Gain Attribute Evaluator with the Ranker method, and results of feature ablation process within 10-CV on train dataset for the selected model; Table S6: Ranking of the feature importance by far transfer/reasoning training using the Information Gain Attribute Evaluator with the Ranker method, and results of the feature ablation process within 10-CV on the train dataset for the selected model; Table S7: Ranking of the feature importance by near transfer/speed-of-processing training using the Information Gain Attribute Evaluator with the Ranker method, and results of the feature ablation process within 10-CV on the train dataset for the selected model; Table S8: Ranking of the feature importance by far transfer/speed-of-processing training using the Information Gain Attribute Evaluator with the Ranker method, and results of the feature ablation process within 10-CV on the train dataset for the selected model; Table S9: Final centroids of K-means clustering for optimal, three-clusters solution with minimum and maximum values across 10 random initializations.
