# Supplementary material for: Machine Learning Approach for Predicting Older Adults’ Responsiveness to Cognitive Training Interventions: Data from the ACTIVE Study
Source: J Intell. 2026 Apr 1;14(4):56. doi: 10.3390/jintelligence14040056 (PMC13117974; doi:10.3390/jintelligence14040056)
Supplement: Supplementary file 1 [file jintelligence-14-00056-s001.zip › jintelligence-4103130-supplementary.pdf]

Supplementary Materials

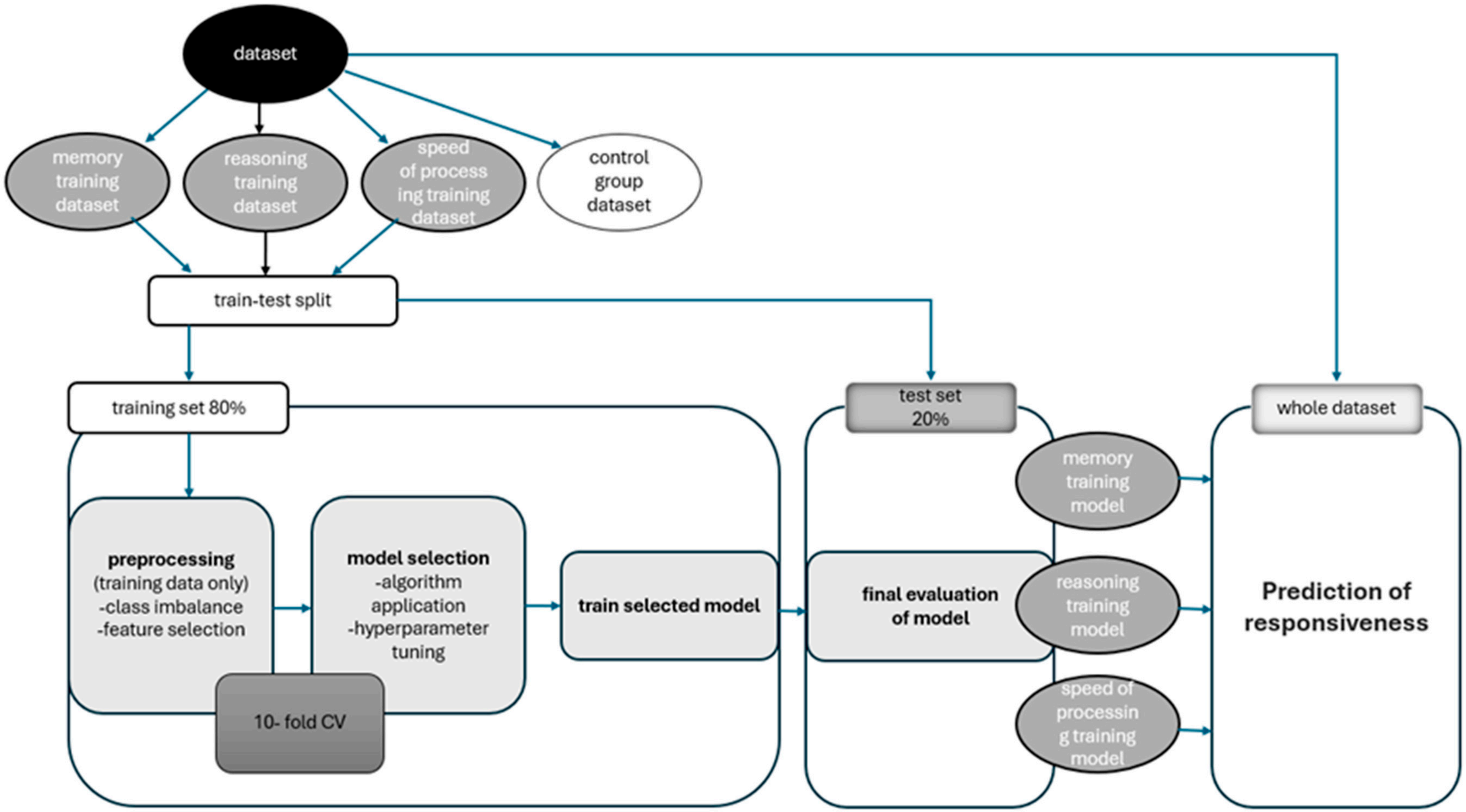

Figure S1. Flowchart of machine learning methodology.

Table S1. RCI calculation results.

	memory training		reasoning training		Speed-of-processing training	
	near transfer					
Negative effect	74	12%	6	1%	10	2%
No effect	409	64%	241	38%	199	31%
Positive effect	158	25%	382	61%	434	67%
Total	641	100%	629	100%	643	100%
	far transfer					
Negative effect	52	8%	120	19%	128	20%
No effect	347	54%	331	53%	338	52%
Positive effect	242	38%	178	28%	187	29%
	641	100%	629	100%	653	100%

Table S2. Correlation matrix of features.

	Feature	1	2	3	4	5	6	7	8	9	10	11	12	13	1[illegible]
1	Age	—	−.039*	−.153**	−.325**	−.369**	−.214**	−.301**	−.288**	−.220**	.207**	.352**	.399**	.285**	−.216[illegible]
2	Education		—	.300**	.226**	.189**	.257**	.381**	.405**	.349**	−.108**	−.181**	−.157**	−.096**	.469*[illegible]
3	MMSE			—	.440**	.368**	.368**	.463**	.462**	.364**	−.180**	−.293**	−.285**	−.167**	.540*[illegible]
4	HVLT				—	.684**	.459**	.496**	.489**	.384**	−.222**	−.409**	−.377**	−.223**	.530*[illegible]
5	AVLT					—	.459**	.435**	.437**	.329**	−.189**	−.365**	−.351**	−.215**	.446*[illegible]
6	Rivermead						—	.453**	.460**	.374**	−.150**	−.293**	−.264**	−.197**	.486*[illegible]
7	Word Series							—	.835**	.625**	−.240**	−.434**	−.432**	−.319**	.649*[illegible]
8	Letter Series								—	.645**	−.231**	−.427**	−.434**	−.314**	.662*[illegible]
9	Letter Sets									—	−.222**	−.353**	−.352**	−.253**	.525*[illegible]
10	UFOV 1										—	.468**	.299**	.154**	−.258[illegible]
11	UFOV 2											—	.566**	.301**	−.434[illegible]
12	UFOV 3												—	.524**	−.375[illegible]
13	UFOV 4													—	−.222[illegible]
14	EPT														—
15	IADL Perf.														
16	IADL Diff.														
17	ADL Perf.														
18	CES-D														

* Correlation is significant at the 0.05 level (2-tailed); ** Correlation is significant at the 0.01 level (2-tailed).

Table S3. Ranking of the feature importance by near transfer/memory training using the Information Gain Attribute Evaluator with the Ranker method, and results of the feature ablation process within 10-CV on the train dataset for the selected model.

Rank (Info Gain Ranker method)	feature	feature ablation step *	ablation step accuracy	ablation step AUC
1	EPT			
2	Years of education	17	0.508	0.572
3	Word Series	16	0.529	0.593
4	Letter Sets	15	0.506	0.596
5	IADL difficulty	14	0.504	0.624
6	CES-D	13	0.508	0.622
7	MMSE	12	0.537	0.624
8	IADL performance	11	0.539	0.616
9	age	10	0.566	0.624
10	ADL performance	9	0.561	0.623
11	HVLT	8	0.568	0.614
12	IMRAW	7	0.590	0.629
13	UFOV 4	6	0.686	0.698
14	AVLT	5	0.668	0.713
15	UFOV 3	4	0.676	0.710
16	UFOV 1	3	0.676	0.712
17	UFOV 2	2	0.676	0.712
18	Gender	1	0.686	0.698

* Additive feature ablation starting from the lowest to higher ranking features.

Abbreviations: EPT= Everyday Problems Test, (I)ADL= (instrumental) activities of daily living, CES-D= Center for Epidemiological Studies—Depression; MMSE= Mini-Mental State Examination; HVLT= Hopkins Verbal Learning Test; IMRAW= Rivermead Behavioral Memory Test immediate recall; AVLT= Rey Auditory–Verbal Learning Test; UFOV= Useful Field of View, subtests 1,2,3 and 4.

Table S4. Ranking of the feature importance by far transfer/memory training using the Information Gain Attribute Evaluator with the Ranker method, and results of the feature ablation process within 10-CV on the train dataset for the selected model.

Rank (Info Gain Ranker method)	feature	feature ablation step *	ablation step accuracy	ablation step AUC
**1**	UFOV 1			
**2**	IADL performance	17	0.627	0.545
**3**	CES-D	16	0.625	0.528
**4**	IADL difficulty	15	0.627	0.554
**5**	MMSE	14	0.625	0.543
**6**	EPT	13	0.625	0.529
**7**	Years of education	12	0.625	0.529
**8**	Age	11	0.623	0.514
**9**	ADL performance	10	0.627	0.507
**10**	HVLT	9	0.623	0.526
**11**	IMRAW	8	0.611	0.524
**12**	Word Series	7	0.607	0.559
**13**	Letter Sets	6	0.609	0.553
**14**	AVLT	5	0.609	0.544
**15**	UFOV_4	4	0.607	0.551
**16**	UFOV_3	3	0.598	0.560
**17**	UFOV_2	2	0.596	0.571
**18**	Gender	1	0.611	0.574

* Additive feature ablation starting from the lowest to higher ranking features.
Abbreviations: EPT= Everyday Problems Test, (I)ADL= (instrumental) activities of daily living, CES-D= Center for Epidemiological Studies—Depression; MMSE= Mini-Mental State Examination; HVLT= Hopkins Verbal Learning Test; IMRAW= Rivermead Behavioral Memory Test immediate recall; AVLT= Rey Auditory–Verbal Learning Test; UFOV= Useful Field of View, subtests 1,2,3 and 4.

Table S5. Ranking of the feature importance by near transfer/reasoning training using the Information Gain Attribute Evaluator with the Ranker method, and results of the feature ablation process within 10-CV on the train dataset for the selected model.

Rank (Info Gain Ranker method)	feature	feature ablation step *	ablation step accuracy	ablation step AUC
1	HVLT			
2	UFOV_2	17	0.602	0.502
3	MMSE	16	0.624	0.535
4	EPT	15	0.630	0.562
5	UFOV_3	14	0.658	0.605
6	UFOV_1	13	0.654	0.606
7	age	12	0.648	0.597
8	IADL difficulty	11	0.654	0.608
9	IADL performance	10	0.648	0.603
10	ADL performance	9	0.650	0.605
11	Years of education	8	0.648	0.602
12	CES-D	7	0.644	0.598
13	IMRAW	6	0.640	0.594
14	Word Series	5	0.646	0.599
15	Letter Sets	4	0.648	0.600
16	AVLT	3	0.646	0.599
17	UFOV_4	2	0.652	0.606
18	Gender	1	0.652	0.606

* Additive feature ablation starting from the lowest to higher ranking features.
Abbreviations: EPT= Everyday Problems Test, (I)ADL= (instrumental) activities of daily living, CES-D= Center for Epidemiological Studies—Depression; MMSE= Mini-Mental State Examination; HVLT= Hopkins Verbal Learning Test; IMRAW= Rivermead Behavioral Memory Test immediate recall; AVLT= Rey Auditory–Verbal Learning Test; UFOV= Useful Field of View, subtests 1,2,3 and 4.

Table S6. Ranking of the feature importance by far transfer/reasoning training using the Information Gain Attribute Evaluator with the Ranker method, and results of the feature ablation process within 10-CV on the train dataset for the selected model.

Rank (Info Gain Ranker method)	feature	feature ablation step *	ablation step accuracy	ablation step AUC
1	UFOV_1			
2	HVLT	17	0.728	0.563
3	UFOV_2	16	0.726	0.580
4	IMRAW	15	0.700	0.590
5	AVLT	14	0.692	0.606
6	UFOV_4	13	0.702	0.623
7	MMSE	12	0.686	0.635
8	Years of education	11	0.672	0.632
9	IADL difficulty	10	0.658	0.649
10	age	9	0.670	0.647
11	IADL performance	8	0.670	0.638
12	CES-D	7	0.674	0.638
13	ADL performance	6	0.684	0.653
14	EPT	5	0.690	0.655
15	Letter Sets	4	0.712	0.678
16	UFOV_3	3	0.700	0.672
17	Word Series	2	0.710	0.674
18	Gender	1	0.698	0.670

* Additive feature ablation starting from the lowest to higher ranking features.

Abbreviations: EPT= Everyday Problems Test, (I)ADL= (instrumental) activities of daily living, CES-D= Center for Epidemiological Studies—Depression; MMSE= Mini-Mental State Examination; HVLT= Hopkins Verbal Learning Test; IMRAW= Rivermead Behavioral Memory Test immediate recall; AVLT= Rey Auditory-Verbal Learning Test; UFOV= Useful Field of View, subtests 1,2,3 and 4.

Table S7. Ranking of the feature importance by near transfer/speed-of-processing training using the Information Gain Attribute Evaluator with the Ranker method, and results of the feature ablation process within 10-CV on the train dataset for the selected model.

Rank (Info Gain Ranker method)	feature	feature ablation step *	ablation step accuracy	ablation step AUC
1	UFOV_4			
2	UFOV_3	17	0.712	0.633
3	UFOV_2	16	0.698	0.718
4	UFOV_1	15	0.706	0.773
5	IMRAW	14	0.708	0.779
6	MMSE	13	0.743	0.802
7	CES-D	12	0.735	0.798
8	IADL difficulty	11	0.745	0.797
9	Years of education	10	0.733	0.790
10	age	9	0.737	0.792
11	IADL performance	8	0.768	0.809
12	HVLT	7	0.763	0.808
13	ADL performance	6	0.765	0.813
14	EPT	5	0.770	0.817
15	Letter Sets	4	0.770	0.814
16	AVLT	3	0.772	0.814
17	Word Series	2	0.780	0.814
18	Gender	1	0.780	0.820

* Additive feature ablation starting from the lowest to higher ranking features.

Abbreviations: EPT= Everyday Problems Test, (I)ADL= (instrumental) activities of daily living, CES-D= Center for Epidemiological Studies—Depression; MMSE= Mini-Mental State Examination; HVLT= Hopkins Verbal Learning Test; IMRAW= Rivermead Behavioral Memory Test immediate recall; AVLT= Rey Auditory–Verbal Learning Test; UFOV= Useful Field of View, subtests 1,2,3 and 4.

Table S8. Ranking of the feature importance by far transfer/speed-of-processing training using the Information Gain Attribute Evaluator with the Ranker method, and results of the feature ablation process within 10-CV on the train dataset for the selected model.

Rank (Info Gain Ranker method)	feature	feature ablation step *	ablation step accuracy	ablation step AUC
1	UFOV_2			
2	EPT	17	0.554	0.597
3	UFOV_3	16	0.559	0.618
4	AVLT	15	0.569	0.624
5	Years of education	14	0.557	0.617
6	IADL difficulty	13	0.552	0.610
7	IADL performance	12	0.565	0.608
8	MMSE	11	0.569	0.612
9	Age	10	0.565	0.609
10	ADL performance	9	0.559	0.603
11	CES-D	8	0.556	0.603
12	HVLT	7	0.554	0.594
13	IMRAW	6	0.552	0.613
14	Word Series	5	0.580	0.635
15	Letter Sets	4	0.590	0.632
16	UFOV_4	3	0.596	0.627
17	UFOV_1	2	0.603	0.625
18	Gender	1	0.592	0.621
* Additive feature ablation starting from the lowest to higher ranking features.				

Abbreviations: EPT= Everyday Problems Test, (I)ADL= (instrumental) activities of daily living, CES-D= Center for Epidemiological Studies—Depression; MMSE= Mini-Mental State Examination; HVLT= Hopkins Verbal Learning Test; IMRAW= Rivermead Behavioral Memory Test immediate recall; AVLT= Rey Auditory–Verbal Learning Test; UFOV= Useful Field of View, subtests 1,2,3 and 4.

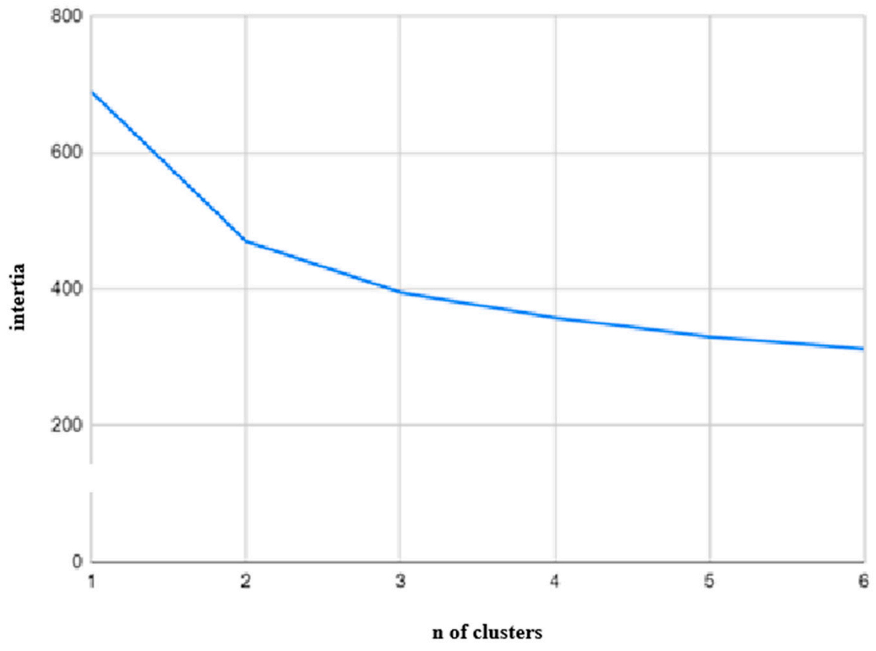

Figure S2. Elbow curve.

Table S9. Final centroids of K-means clustering for optimal, three-clusters solution with minimum and maximum values across 10 random initializations.

	Attribute	full data	High cognitive functioning (cluster 1) (min-max)	Average functioning (cluster 2) (min-max)	Low cognitive functioning (cluster 3) (min-max)
Ability	Test	n=2802	n=948 (948–958)	n=1259 (1259–1270)	n=595 (574–595)
Baseline memory	HVLT	−0,028	0,672(0,669–0,672)	−0,171(−0,186–−0,171)	−0,843(−0,846–−0,843)
	AVLT	−0,029	0,640(0,635–0,640)	−0,181(−0,193–−0,181)	−0,772(−0,782–−0,772)
	RVM	−0,0013	0,766(0,759–0,766)	−0,304(−0,315–−0,304)	−0,584(−0,584–−0,578)
Baseline reasoning	WS	−0,001	0,955(0,950–0,955)	−0,348(−0,361–−0,348)	−0,787(−0,789–−0,787)
	LT	−0,009	0,870(0,865–0,870)	−0,340(−0,355–−0,340)	−0,710(−0,710–−0,703)
Baseline speed of processing	UFOV1	0,000	−0,280(−0,280–−0,277)	−0,153(−0,153–−0,150)	0,781(0,781–0,797)
	UFOV2	−0,008	−0,584(−0,584–−0,583)	−0,276(−0,276–−0,265)	1,537(1,537–1,557)
	UFOV3	−0,001	−0,705(−0,705–−0,700)	0,024(0,024–0,031)	1,099(1,099–1,106)
	UFOV4	0,001	−0,522(−0,522–−0,512)	0,130(0,128–0,130)	0,571(0,571–0,572)

Abbreviations: HVLT= Hopkins Verbal Learning Test; AVLT= Rey Auditory–Verbal Learning Test; RVM= Rivermead Behavioral Memory Test immediate recall; WS= Word Series, LT= Letter Sets; UFOV= Useful Field of View, subtests 1,2,3 and 4.
